# Importance of Interface in the Coarse-Grained Model of CNT /Epoxy Nanocomposites

**DOI:** 10.3390/nano9101479

**Published:** 2019-10-17

**Authors:** Ke Duan, Li Li, Fei Wang, Weishuang Meng, Yujin Hu, Xuelin Wang

**Affiliations:** 1 State Key Lab of Digital Manufacturing Equipment and Technology, School of Mechanical Science and Engineering, Huazhong University of Science and Technology, Wuhan 430074, China; duank@hust.edu.cn (K.D.); d201880192@hust.edu.cn (F.W.); m201770371@hust.edu.cn (W.M.); wangxl@hust.edu.cn (X.W.); 2 School of Mechanical and Aerospace Engineering, Nanyang Technological University, Nanyang Avenue, Singapore 639798, Singapore; lili_em@hust.edu.cn

**Keywords:** interface force fields, CNTs/epoxy nanocomposites, coarse-grained model, molecular dynamics

## Abstract

Interface interactions play a crucial role in determining the thermomechanical properties of carbon nanotubes (CNTs)/polymer nanocomposites. They are, however, poorly treated in the current multi-scale coarse-grained (CG) models. To develop suitable CG models of CNTs/polymer nanocomposites, we demonstrate the importance of two aspects for the first time, that is, preserving the interfacial cohesive energy and reproducing the interface load transfer behavior of all-atomistic (AA) systems. Our simulation results indicate that, for CNTs/polymer nanocomposites, the interface cohesive energy and the interface load transfer of CG models are generally inconsistent with their AA counterparts, revealing significant deviations in their predicted mechanical properties. Fortunately, such inconsistency can be “corrected” by phenomenologically adjusting the cohesive interaction strength parameter of the interface LJ potentials in conjunction with choosing a reasonable degree of coarse-graining of incorporated CNTs. We believe that the problem studied here is general for the development of the CG models of nanocomposites, and the proposed strategy used in present work may be applied to polymer nanocomposites reinforced by other nanofillers.

## 1. Introduction

During the past few decades, polymer nanocomposites (PNCs) have received significant attention and have long been at the forefront of research in the polymer community for their broad range of potential applications [1,2,3,4,5,6,7]. PNCs, different from traditional composite materials, exhibit quite complex thermomechanical properties which are strongly related to the physics and chemistry of the inclusion of nanofillers, including nanoparticles, carbon nanotubes (CNTs) [4,8], and graphenes [9,10]. Among all PNCs, the most attractive materials may be those reinforced by carbon nanotubes, due to their practical engineering applications such as aerospace materials, packing, and electromagnetic interference (EMI) shielding [4,11,12].

Although very promising, most of the practical applications of CNTs-reinforced PNCs are currently hindered by the lack of comprehensive understanding of their microstructure-to-property relationships [5,13]. As widely demonstrated, the reinforcing efficiency of CNTs in the mechanical properties of PNCs is highly dependent on several factors such as dispersion state of CNTs [14,15], CNTs waviness and orientations [16], and surface functionalization [17]. In general, it is extremely challenging to control and measure these factors in experiments, and their influences on the mechanical properties of CNTs-reinforced PNCs can only be studied via a variety of computational modeling approaches [18,19]. Overall, these computational methods can be divided into three categories—(1) molecular- or micro-scale methods (such as molecular dynamics (MD) and Monte Carlo simulations), (2) meso-scale methods exemplified by dissipative particle dynamics (DPD) and Brownian dynamics, and (3) macro-scale methods (e.g., equivalent-continuum, and finite element method) [18]. Among these numerical approaches, MD simulations are an extremely powerful tool due to their capability to provide realistic interactions between CNTs and polymer-matrix. The interactions are, however, typically approximated or poorly treated in other methods. Such advantage enables MD simulations to probe interfacial phenomena occurring at nanoscale, such as stick-slip damping mechanism [20], and theoretically to consider any structural characteristics of CNTs (dispersion state, waviness, orientations and so on) for studying the mechanical properties of PNCs. The computational efforts of using all-atomic MD simulations to establish the desired structural-properties relationships of CNTs-reinforced PNCs have, however, been limited by the massive computational resources needed which are typically beyond the ability of modern facilities.

Alternatively, developing coarse-grained (CG) models that are capable of preserving the mechanical properties of their all-atomistic (AA) counterparts has been an attractive approach for providing fundamental insights into the mechanical behaviors of polymers or their nanocomposites [21,22,23]. In comparison with AA simulations, the accessible time and length scales for CG models are substantially increased by removing the “unessential” atomistic features within molecules and thus significantly reducing the number of degrees of freedom to obtain the properties of interest [24]. Several approaches have been proposed to derive the force fields of CG models from their AA counterparts, such as iterative Boltzmann inversion (IBI) method [25], force matching [26,27], and inverse Monte Carlo methods [28]. As for the CNTs-reinforced PNCs, although the force fields of CNTs and polymer matrix can be successfully derived, respectively, from the force matching and IBI approach [23,29,30], there is still a lack of the definition on the interface interactions between CNTs and the polymer matrix material. To the best of the authors’ knowledge, only a few studies are devoted to the development of the CG models of CNTs-reinforced PNCs [21,31,32]. In these studies, the CNTs/polymer interface was simply described by Lennard-Jones (LJ) potentials where the key parameters of interfacial LJ pairs are directly predicted by using an arithmetic mean manner without deep theoretical support. Furthermore, the degree of coarse-graining for CNTs was chosen for the CG models of CNTs-reinforced PNCs without any evaluation on its reasonability [21,32]. As a result, the interfacial characteristics of CNT-reinforced PNCs predicted the AA and CG models might be significantly deviated, leading to unforeseen artifacts in the mechanical properties obtained from the CG models.

In the present study, we aim to provide insights into the problem of developing a reasonable CG model for CNTs-reinforced PNCs. Specific attention is focused on the comparison of the interface characteristics between AA and CG models. We have two key findings—(1) the interfacial cohesive energy across the CNTs/epoxy interface modeled using CG models, which is described by LJ potentials similar to the previous researches, is significantly overestimated compared with its real AA counterpart; and (2) the adopted degree of coarse-graining λ for CNT is found to play a critical role in affecting the interfacial shear behavior, emphasizing the importance of evaluating the reasonability of the adopted λ and the interfacial force fields before using the CG models to probe mechanical properties.

## 2. Simulation Methods

### 2.1. Potentials and Molecular Models

Many universal force fields have been developed for describing the interactions of AA models, such as Dreiding [33], consistent valence force field (CVFF) [34], and polymer consistent force field (PCFF) [35]. CG potentials are, however, not unique, even for a specific material. In fact, they are highly dependent on many factors, such as the coarse-graining scheme and the thermodynamic state of CG models (temperature and pressure) [23]. The main purpose of this work is to provide an insight into the development of a reasonable CG model considering carefully the crucial role of interface on the mechanical properties of CNT/epoxy nanocomposites. To achieve this, we chose a typical CNT/epoxy nanocomposite in which (5,5) CNT was considered as the reinforcement, and a commonly used engineering cross-linked system which composed of diglycidyl ether of bisphenol A (DGEBA) and 4,4-diaminodiphenyl methane (DDM) was selected as the matrix.

#### 2.1.1. All-Atomic Model

**Figure 1:**
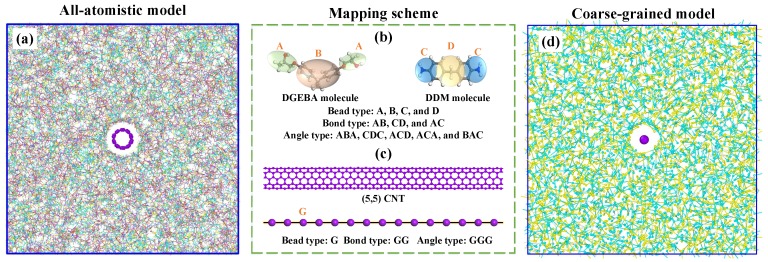
Schematic view of mapping all-atomistic model to coarse-grained model for epoxy nanocomposites reinforced by (5,5) carbon nanotube (CNT). (**a**) Snapshot of the all-atomistic model, (**b**) the adopted mapping scheme for DGEBA and DDM molecules, (**c**) the adopted mapping scheme for (5,5) CNT, and (**d**) snapshot of the resulting coarse-grained model.

The initial nanocomposite model, as presented in Figure 1a, was constructed with two procedures. First, the 75.7 Å long (5,5) CNT (periodically in the longitudinal direction) was placed at the center of a simulation unit box. Then, the DGEBA and DDM molecules (there are 574 and 287 molecules for DGEBA and DDM, respectively.) were packed into the extra space of the simulation box with a Monte Carlo style similar to our previous work [2]. The general-purpose Dreiding force field was adopted to describe the interactions between atoms, in combination with long-range Columbic interactions where atomic partial charges were determined by the charge equilibration (QEq) method [33,36]. The used cutoff distance for van der Waals and electrostatic interactions was 12 Å. To relax the initial simulation model, an equilibration procedures similar to our previous work was carried out [23,37]. After that, the fully relaxed nanocomposite model was cross-linked to a specific degree of cross-linking (DOC) at temperature 700 K using a dynamic cross-linking strategy [37]. The details for the adopted dynamics cross-linking strategy can be found in our previous paper [37]. Finally, the cross-linked nanocomposite model was again equilibrated to a target temperature. For more details about the constructed model and equilibration procedures, please refer to Supplementary Section S1.1. Periodic boundary conditions were imposed on all three directions of the simulation cell, namely, the *x*, *y*, and *z* directions. A timestep 1 fs was used for all AA simulations.

#### 2.1.2. Coarse-Grained Model

The construction and equilibration processes of the CG models for CNT/epoxy nanocomposites are similar to those of their AA counterparts and we present these technical details in Supplementary Section S1.2. In this section, we are mainly concerned with the issue of force fields used in the CG model of CNT/epoxy nanocomposites. For the CNT/epoxy nanocomposites, the force fields can be divided into three parts—(1) reinforcement force fields, that is, the interactions for particles in one (5,5) CNT or between different (5,5) CNTs, (2) matrix force fields, namely both the intra- and inter-molecule interactions for DGEBA and DDM molecules, (3) interfacial force fields between the (5,5) CNT and the cross-linked epoxy matrix.

For CNTs, the fundamental mechanical parameters of their CG models can be derived from AA simulations based on the matching of energies and mechanical features, including tensile stiffness, bending stiffness and adhesion properties. Specifically, the force field of (5,5) CNT used here has been already reported by Buehler [29] and was widely-adopted for investigating the tensile and viscoelastic properties of CNT-based materials [38,39,40]. As for the cross-linked epoxy matrix studied here, the coarse-grained force fields were determined through a combination of the IBI method and the machine-learning based approach, as reported in our previous work [23]. The mapping scheme for DGEBA and DDM molecules are the same as that in our previous work [23], illustrated in Figure 1b. The IBI method was used to preserve the structural aspect of the matrix molecule and thus determine the bonded interaction parameters (including the bond and angle potentials). By using a robust machine-learning based technique, we determined the nonbonded interaction (the 12-6 LJ potential form) to reproduce the density, glass transition temperature and elastic moduli of cross-linked epoxy at a wide range of temperatures and various values of DOC. The main feature of the nonbonded potential is that the strength parameter of the cohesive interaction ϵ was DOC- and temperature-dependent. The CG model for cross-linked epoxy has four types of CG beads (beads ‘A’, ‘B’, ‘C’, and ‘D’) and the detailed force fields are presented in Supplementary Section S2.1.

Since the CG potentials of the CNT and cross-linked epoxy were derived separately by means of totally different approaches, the determination of the CG potentials of CNT/epoxy nanocomposites requires an additional definition of the CNT/epoxy interface interaction. Beside, the degree of coarse-graining λ for CNT may have a significant effect on the interfacial interactions and thus on the mechanical properties of the nanocomposites due to the fact that a large λ typically leads to artificial surface roughness [41]. However, to the best of our knowledge, there is nearly no previous literature considering the crucial issue whether the adopted degree of coarse-graining λ for CNT is reasonable for studying the problems at hand?

With the above question in mind, the CG models of the CNT/epoxy nanocomposites were constructed in such a way that the degree of coarse-graining λ can be adjusted from 4 Å to 10 Å. Figure 1c depicts the mapping scheme of (5,5) CNT using λ = 5 Å. The bead within a single CNT was labeled as ‘G’, forming 1 bond type ‘G G’ and 1 angle type ‘G G G’. The force fields of (5,5) CNT with various values of λ can be found in Supplementary Section S2.2. Based on the mapping rules described above, a snapshot of the CG models of CNT/epoxy nanocomposites, in which epoxies are not cross-linked yet, is shown in Figure 1d. For all CG simulations, timestep is 4 fs unless otherwise mentioned. All the CG and AA simulations were performed with large-scale atomic/molecular massively parallel simulator (LAMMPS) [42] and visualized via the Open Visualization Tool (OVITO) [43].

### 2.2. Evaluation of the Interface Reasonability

As is widely-known, the key feature of nano-reinforcements is the extremely larger contact surface area with polymer-matrix in comparison with traditional fillers, enabling enormous interesting properties of the resulted nanocomposites [5,44]. Therefore, the load transfer behavior of the CNT/epoxy interface should be well preserved under coarse-graining, otherwise the developed CG models may lead to unforeseen artifacts on the studied properties. Essentially, the load transfer of a CNT/epoxy interface can be characterized by the force-separation responses in radial opening and axial sliding mode separations [45,46,47]. Similarly, we here use this approach to evaluate the interface behavior of the coarse-grained CNT/epoxy interface which is compared to their AA counterpart. Specifically, we are more concerned with the equivalence issue of CNT/epoxy interface between the AA and CG models. To accompany this, we adopt a strategy called as “monomeric separation response” which is done by performing radial opening and sliding mode separation simulations of a single matrix molecule onto the CNT surface and recording the force-separation response, as illustrated in Section 2.2.2. As demonstrated by previous research, the interfacial strength of the CNT/polymer interface is typically an additive effect of the monomeric friction [48]. Thus, it is feasible to use the “monomeric separation response” approach for evaluating the interface load transfer behavior for the CNT/epoxy nanocomposites here.

#### 2.2.1. Calculation of the Interfacial Cohesive Energy

Before illustrating the strategy of “monomeric separation response,” it is necessary to pay attention to the interface cohesive energy, which determines the traction force significantly for both radial opening and axial sliding separation. Thus, the first thing to be satisfied is an identical interfacial cohesive energy between AA and CG models, otherwise the evaluation for load transfer of the CNT/epoxy interface has no meaning.

The total energy of a CNT/epoxy nanocomposite unit cell can be expressed as

(1) $$\begin{array}{c} E_{total}=E_{CNT}+E_{epoxy}+E_{interface} \end{array}$$

where $E_{total}$ indicates the total energy of the simulation system, $E_{CNT}$ represents the energy of embedded CNTs, $E_{epoxy}$ is the energy of the epoxy matrix, and $E_{interface}$ represents the energy of the CNT/epoxy interface. The interfacial cohesive energy per unit length can then be calculated as [49]

(2) $$\begin{array}{c} \gamma =\frac{E_{\mathrm{int}erface}}{2A} \end{array}$$

where *A* is the contact area of the interface, determined as A=πdL where *d* and *L* are the diameter (6.78 Å) and the embedded length of the used (5,5) CNT, respectively. It should be mentioned that the value of *L* is determined to be the dimension of simulation box at a full equilibrated state along the longitudinal direction of the (5,5) CNT.

#### 2.2.2. Characterization of the Interface Load Transfer

**Figure 2:**
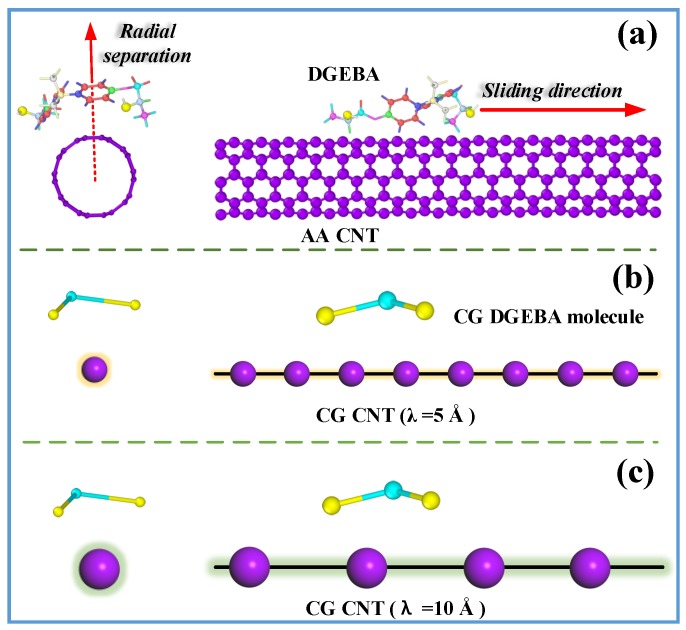
Illustration of adopted strategy “monomer separation response” for evaluating the interface load transfer of CNT/epoxy nanocomposites: (**a**) all-atomistic (AA) system, (**b**) coarse grained (CG) model with λ = 5 Å, and (**c**) CG model with λ = 10 Å. The “monomer separation response” includes radial opening simulation which is done by separating a DGEBA molecule from the (5,5) CNT along its radial direction, and axial sliding mode separation simulation which is done by forcing the DGEBA molecule slides on the CNT surface along its axial direction.

The behavior of the CNT/epoxy interface is evaluated by the force-separation responses in axial opening and sliding mode separations, as shown in Figure 2. For the AA system, as depicted in Figure 2a, a fully equilibrated DGEBA molecule was forced to separate from the (5,5) CNT along the radial direction for the radial opening mode, and to slide on the CNT surface along its axial direction for the sliding mode separation. For the CG models, the simulation procedures are the same, as done for their AA counterparts. As mentioned in Section 2.1.2, λ can be changed and then leads to totally different CG force fields. Thus, it is of great significance to examine whether λ has any effects on the interface behavior. In this work, 7 different λ values ranging from 4 to 10 Å were considered. Figure 2b,c illustrated the schematic view of “monomeric separation response” for CG interface models with λ being 5 and 10 Å, respectively.

## 3. Results And Discussion

### 3.1. Preserve the Interface Cohesive Energies

As described in Section 2.2.1, the interface cohesive energy is a dominant factor in affecting the load transfer across the interface. Thus, the AA interface cohesive energy between CNT and epoxy matrix should be examined and preserved before evaluating the equivalence issue of the interface load transfer between the AA and CG models. To achieve this goal, we constructed 8 simulation models for the CNT/epoxy nanocomposites, including 1 AA (Figure 1a) and 7 CG models (an example is shown in Figure 1d). The degree of cross-linking for all these 8 models are kept the same value of 90%. The target temperature to extract the interface cohesive energy of various simulation models was 300 K. The CNT is periodical along its longitudinal direction to eliminate the end edge interaction effects, in order to determine an accurate value of the interface cohesive energy.

**Figure 3:**
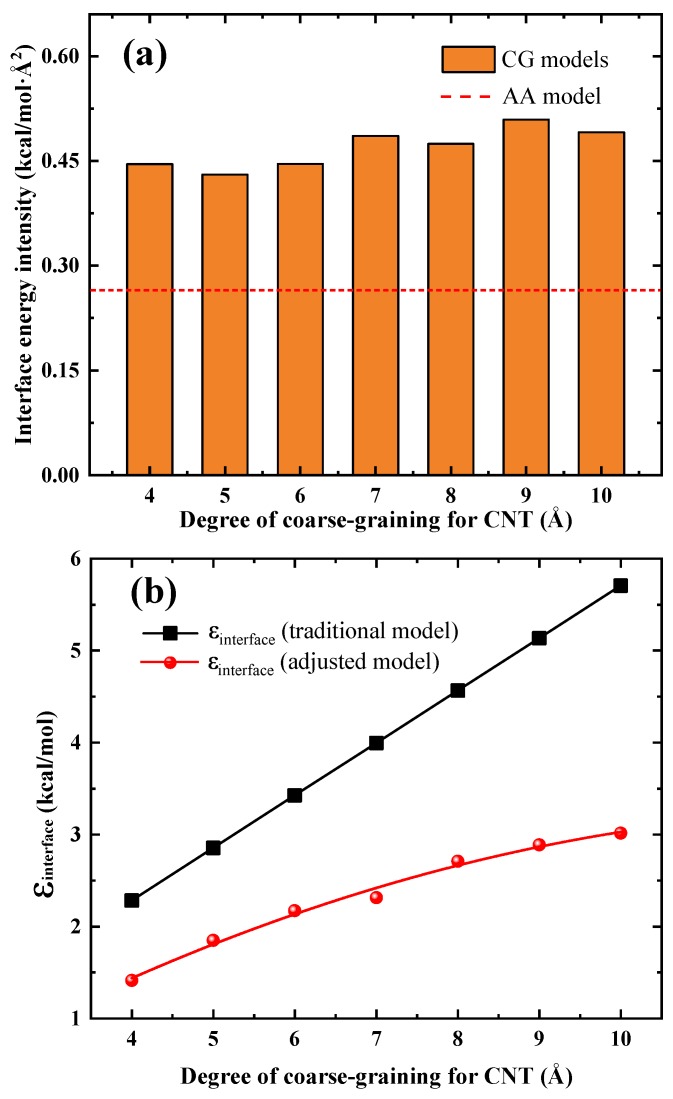
(**a**) Comparison of the interface cohesive energies between the AA and CG models, and (**b**) $\varepsilon_{\mathrm{interface}}$ vs. λ curves for the traditional interface model (black line) and the adjusted interface mod el (red line) in this work.

Figure 3a shows the comparison of the interface cohesive energy for both the AA (red dash line) model and the CG models with different values of λ. The obtained interface cohesive energy for all the CG models is not equal to that of the AA model. In fact, the interface cohesive energy of any CG model is significantly higher than that of its AA counterpart. This phenomenon strongly emphasizes the fact that well description of the interactions for each constitutive law of the phases in nanocomposites does not imply the correctness of the whole nanocomposite model. In other words, the interface interactions between two components may be significantly altered when developing a coarse-grained model of a AA system. Another thing can be observed from Figure 3 is that there is no apparent trend between the interfacial energy (CG models) and the degree of coarse-graining λ. This is mainly due to the reason that the interaction cohesive strength ϵ increases linearly (see black line of Figure 3b with the degree of coarse-graining λ, however, the number of vdWl pairs decrease linearly with λ (under the ideal conditions). For instance, Figure 2c presents the condition where the λ is two times that of the condition shown in Figure 2b. Since the length scale parameter σ (see Equation (3)) is independent of λ, the distributed state of matrix beads is expected to be the same for the two cases under ideal conditions. Thus, the number of vdWl pairs for condition shown in Figure 2c is only one half that for condition shown in Figure 2b. Considering the fact that the energy of single vdWl pair for Figure 2c is two times that of for Figure 2a, we can conclude that the interfacial energy for the two cases is the same under ideal conditions. However, for the real CG simulations the distribution state of matrix beads will be affected by not only the initial sampling of the matrix molecules but also the varied λ (not significantly), leading to the fluctuation of interfacial energy of CG models at different degree of coarse-graining λ.

To understand the observed inconsistency of interface cohesive energy between AA and CG models, the key factor to be considered is the used nonbonded potentials for describing the interface. For the nanocomposites studied here, the nonbonded interactions for both AA and CG models are modeled using 12-6 LJ potential (as mentioned in Section 2), having an energy expression:

(3) $$\begin{array}{c} E_{LJ}=4\varepsilon \left[{\left(\frac{\sigma }{r}\right)}^{12}-{\left(\frac{\sigma }{r}\right)}^{6}\right] \end{array}$$

where *r* represents the distance between AA atoms or CG particles, ε indicates the depth of the potential well, and σ indicates the radial distance at which the inter-particle potential energy is zero. The detailed values of ε and σ for epoxy and CNT particles are presented in Supplementary Section S2. As for the interface cohesive energy of CG models, they are contributed by 4 interface LJ pairs, that is, GA, GB, GC, and GD. For a traditional manner, the parameters of these 4 LJ pairs are typically determined by the following forms, as done by previous papers [21,31,32].

(4) $$\varepsilon_{ij}=\sqrt{\varepsilon_{ii}\varepsilon_{jj}},\;\sigma_{ij}=\left(\sigma_{ii}+\sigma_{jj}\right)/2$$

in which subscript *i* represents the CNT particle ’G’, and subscript *j* denotes different particle types of epoxy (particle type A, B, C, and D). In this manner, the cohesive interaction strength parameters $\varepsilon_{interface}$ as a function of λ of these CG models are shown in the black line of Figure 3b. It should be clarified here that the value of $\varepsilon_{ij}$ is identical for the 4 LJ pairs (GA, GB, GC, and GD) which contribute to all the interface van der Waals (vdWl) energies and we name this unified value $\varepsilon_{interface}$. This is because all epoxies particle types (particle type A, B, C, and D, as shown in Figure 1b share an identical value of ε (we refer the reader to our previous paper [23] for detailed reasons).

**Figure 4:**
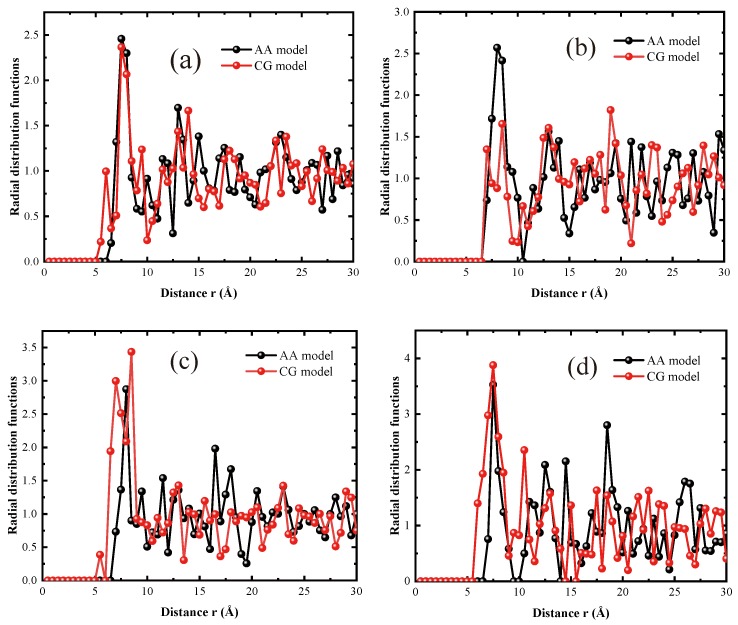
Radial distribution function (RDF) curves for (**a**) particle type ‘A’, (**b**) particle type ‘B’, (**c**) particle type ‘C’, and (**d**) particle type ‘D’.

Since both $\varepsilon_{interface}$ and σ of the interfacial LJ interactions are critical for contributing to the interface cohesive energy of the CG models, we separately examined whether their characteristics are well captured by the CG models. We proceeded to evaluate the reasonability of length scale parameter σ of the 4 interface LJ pairs ($\sigma_{GA}$, $\sigma_{GB}$, $\sigma_{GC}$, and $\sigma_{GD}$) for CG models. This is done by comparing the interfacial radial distribution function (RDF) curves for the AA and CG models. Note that the RDF curves characterize the probability of locating epoxy molecules in a surface of CNT at the distance *r*, and thus characterize the situation of the effective zone of the particles that contribute to the interfacial cohesive energy. We selected a maximum *r* = 30 Å and divided it into 60 small sections to determine the RDF curves (please refer to Supplementary Section S3 for more details). It should be noted that we chosen the maximum *r* to be 30 Å because this distance is equal to the cutoff distance for the van der Waals force of the CG models. Beyond this distance, the interactions between CNT and epoxy molecules are negligible. The comparison of the RDF curves for the four types of matrix beads between the AA and CG models is shown in Figure 4. It can be observed that, although the RDF curves for both the AA and CG models exhibit relatively high fluctuation, their RDF curves are generally in good agreement with each other. The relatively high fluctuation is mainly attributed to the lack of movement of matrix particles because of the high degree of cross-linking (DOC) and the glassy state (the glass transition temperature for DOC-90 cross-linked epoxy is ∼437.32 K) [23]. The consistent RDF curves imply that the distributing state of epoxy molecules around the CNT surface of the AA model can be faithfully described by the CG model in which the length scale parameter σ of the 4 interface LJ pairs are determined by Equation (4). Thus, the overestimated interface cohesive energies in the CG models mainly originate from the cohesive interaction strength parameter $\varepsilon_{interface}$ used for modeling the interfacial LJ interactions.

To preserve the interface cohesive energy of the AA models, we phenomenologically adjust $\varepsilon_{interface}$ for CG models with different values of λ. The adjusted value of $\varepsilon_{interface}$ as a function of λ is shown in the red line of Figure 3b. It is clear that $\varepsilon_{interface}$, which preserves the AA interface cohesive energy for CG models, is much smaller than that predicted by traditional model (black line) using Equation (4), emphasizing the importance of carefully re-adjusting the interface interactions when modeling the nanocomposites via CG models. The trend of $\epsilon_{interface}$ with the degree of coarse-graining λ can be fit with a continuous quadratic polynomial function of the form $\varepsilon_{interface}=a\lambda^{2}+b\lambda +c$, where a = −0.02075 kcal/molÅ${}^{2}$, b = 0.556 kcal/molÅ, and c = −0.45265 kcal/mol. This relationship can be used to determine the value of $\epsilon_{interface}$ for a specific adopted λ within the range of 4 to 10 Å which has not given explicitly by our simulations.

### 3.2. Effects of λ on the Interfacial Load Transfer

In Section 3.1, we preserved the interfacial cohesive energy of the AA models by phenomenologically adjusting the cohesive interaction strength parameter $\varepsilon_{interface}$ of the CG models. In this section, we turn our focus on evaluating the equivalence issue of interface load transfer between the AA and CG models using the “monomeric separation response” approach. It should be noted that the interface energy of the CG models in this section is consistent with their AA counterparts by using the phenomenologically determined $\epsilon_{interface}$ in Section 3.1 to adjust the interface cohesive energy of the CG models.

As described in Section 2.2.2, 7 different values of λ were considered. Thus, there are totally 8 interface models including 1 AA and 7 CG interface models. For each model, a DGEBA molecule was fully equilibrated on the CNT surface and subjected to radial and sliding separations to obtain the force-separation responses using two separation simulations.

For the sake of convenience, we designated these CG interface models in terms of “λ-*d*” terminology where *d* is equal to the equilibrium distance between two CNT beads, characterizing the degree of coarse-graining, and indicates the actual value of λ.

#### 3.2.1. Separation Force Due to Radial Opening Mode

**Figure 5:**
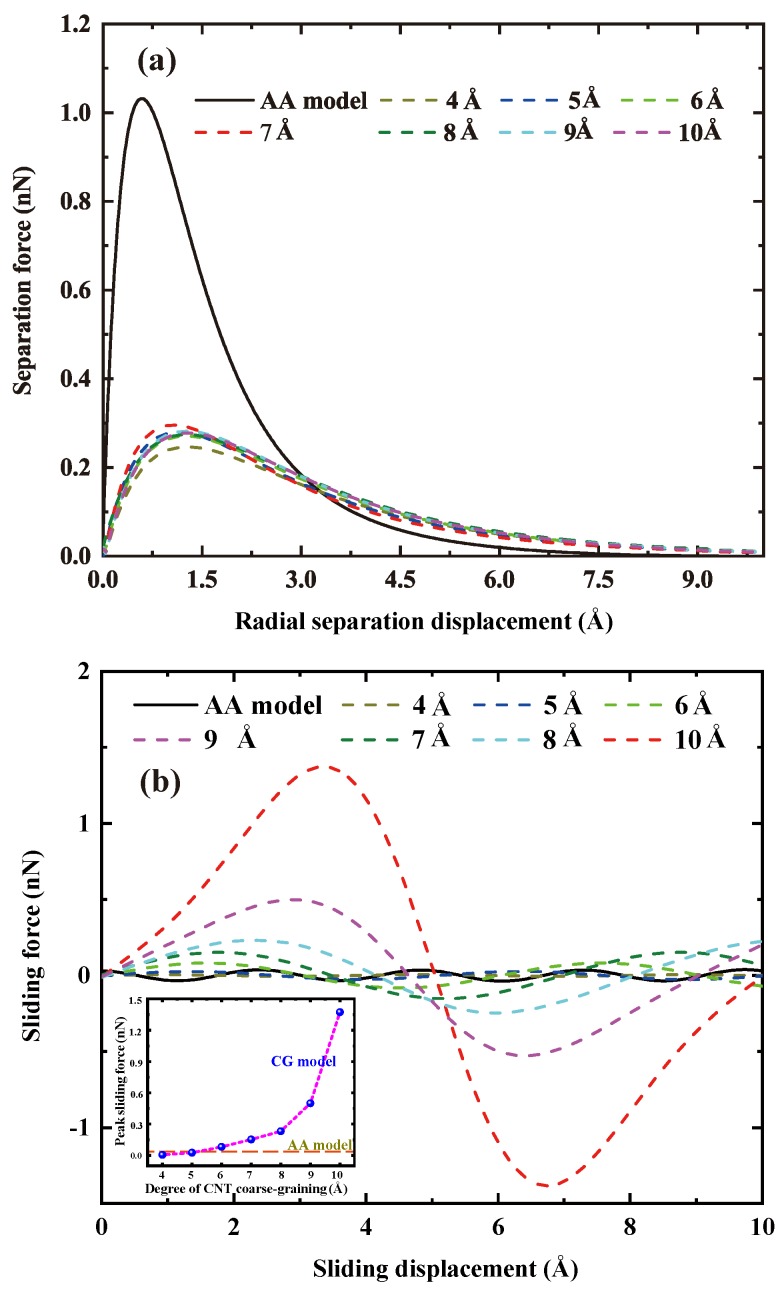
Force-separation curves of the “monomeric separation response” for the AA and CG interface models, (**a**) radial opening mode, and (**b**) axial sliding mode.

Figure 5a illustrates the traction force of the DGEBA molecule for both the AA (black solid line) interface model and the 7 CG interface models (dash lines). All the force-separation curves exhibit a similar pattern in which the traction force initially increases rapidly to peak force and then gradually decreases to zero with the increasing separation displacement, which is typical for vdWl-dominated interface [45,46]. Surprisingly, the peak traction force for all these CG interface models is, however, much smaller than that of their AA counterparts, implying that the interface load transfer in the CNT radial directions is underestimated by the CG models. As observed from Figure 5a, the peak traction force for the CG models is around 0.28 nN, which is less than one thirds that of the AA model (∼1.03 nN). The force inconsistency between the AA and CG models indicates that the CG model cannot simultaneously preserve both the force (radial traction force here and sliding force in Section 3.2.2) and the interfacial energy, which has also been observed in other literature [50,51]. This phenomenon is mainly due to the shift between enthalpy and entropy upon coarse-graining AA models [50,51,52]. As for these CG models, no evident dependence is observed between the studied λ and the resulted radial traction forces.

#### 3.2.2. Sliding Force Due to Axial Sliding Mode

Force-separation responses for the axial sliding mode are shown in Figure 5b, all of which exhibit a sinusoidal-alike pattern which arises from the periodic crystal structure of both the AA and CG CNT models along their longitudinal direction. The periodic distance is equal to λ for the CG models (Figure 2b,c), while is the distance between two neighboring hexagonal lattice centers along the longitudinal direction for AA CNT (∼2.5 Å, Figure 2a). For instance, the red dash curve in Figure 5b shows a periodic 10 Å for the λ-10 CG interface model.

Aside from the sinusoidal-alike pattern of these force-separation curves, a very important observation in Figure 5b is the difference in peak sliding forces between the AA and CG models. As clearly illustrated in Figure 5b, the peak sliding force of CG models is strongly dependent on the adopted λ, exhibiting a nonlinear fashion as demonstrated by the inset of Figure 5b. Specifically, the peak sliding force increases slowly when the adopted λ is less than 8 Å, whereas shows a drastic increase when λ is beyond 8 Å. As a comparison, the required peak sliding force is 1.37 nN for the λ-10 interface model, which is almost 3 orders of magnitude higher than that of the λ-4 model (0.0054 nN). The real peak sliding force derived from the AA model is about 0.0354 nN (the black solid line in Figure 5b), which is much less than that of the studied CG models. From the sliding mode separation characteristic point of view, we can conclude that in order to capture the AA sliding characteristics reasonably, there is a limit of the degree of coarse-graining λ of the CNT beyond which the mechanical properties of the CG model of the CNT are too deteriorated. As seen, the interface appears to be too rough if λ is too large and too smooth if λ is too small. Here, the suitable λ of (5,5) CNT is around 5 Å due to the reason that the peak sliding force of the λ-5 interface model (0.026 nN) is the closest to that of the AA model (0.0354 nN), as suggested by the inset of Figure 5b.

### 3.3. Effect of λ on Elastic Moduli

As illustrated in Section 3.2, the interface load transfer behavior of CG models is typically different to their AA counterparts and is, in fact, highly dependent on λ. As a result, implementing different values of λ into the CG models may lead to seriously unforeseen artifacts on the predicted mechanical properties of the CNT/epoxy nanocomposites. To justify this, we investigated a simple but representative problem right here for CNT/epoxy nanocomposites, namely the effect of the CNT’s length on the tensile moduli of CNT/epoxy nanocomposites.

The CG models were constructed in such a way that 4 parallel coarse-grained (5,5) CNT with their axes along the same direction (which is called the longitudinal direction of the nanocomposites) were fully embedded into the epoxy-matrix. Furthermore, we name the other two directions as the transverse (or lateral) directions.

**Figure 6:**
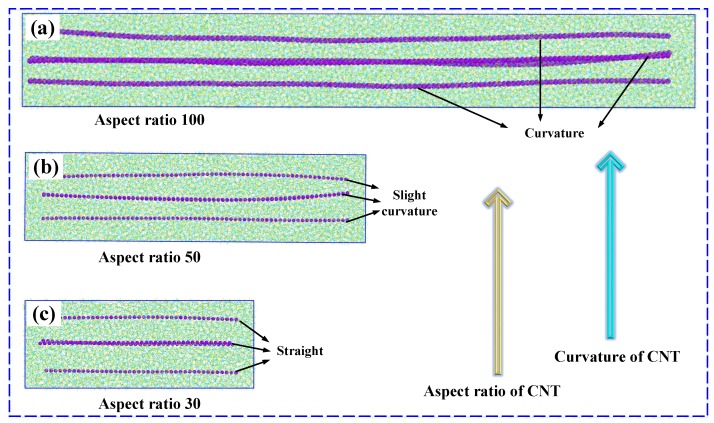
Fully relaxed configurations of the CG models of epoxy-matrix nanocomposites, each of whose cells have 4 embedded CNTs, (**a**) the aspect ratio of CNTs is 100, (**b**) the aspect ratio of CNTs is 50, and (**c**) the aspect ratio of CNTs is 30. All the degrees of coarse-graining of the CNTs are chosen as λ=5 Å.

Figure 6 shows parts of the fully relaxed configurations of the CG models of epoxy-matrix nanocomposites, each of whose cells has 4 embedded CNTs. When modeling these CNT/epoxy nanocomposites, 4 different values of λ were considered, that is, λ = 4, 5, 6 and 10 Å. For each studied λ, 6 different length-to-diameter ratios, ranging from 10 to 150, were considered.

To differentiate these constructed CG models, we use a terminology of “λ-*d*-α” where *d* indicates the actual value of the degree of coarse-graining of the CNT, as defined above, and α represents the specific value of the aspect ratio of the embedded CNT. For all the CG models, the mass fraction of CNTs was kept the same at 5% to eliminate the influence of CNT contents.

**Figure 7:**
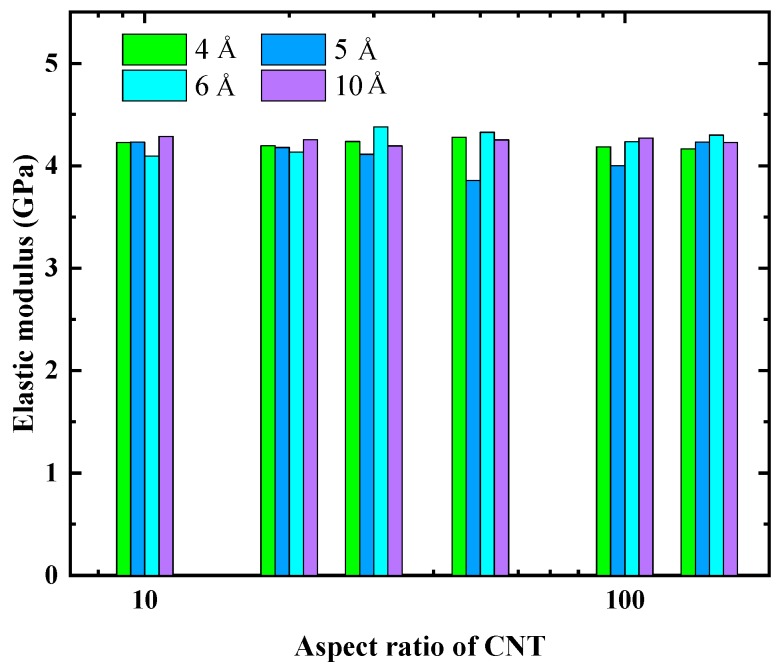
Averaged lateral elastic moduli of epoxy-matrix nanocomposites reinforced by the CNTs which have different aspect ratios and various degrees of coarse-graining.

We proceeded to evaluate the influence of λ on the elastic responses of nanocomposites in the two transverse directions. Figure 7 shows the comparison of the averaged lateral elastic modulus for these CG models. It is clear that the lateral elastic modulus for all these CG models are comparable to each other, yielding values of around 4.2 GPa. Specifically, the value is also comparable to the elastic modulus of the epoxy matrix (∼3.97 GPa), implying that the embedded CNTs show a negligible improvement on the lateral elastic modulus of the CNT/epoxy nanocomposites. This phenomenon is consistent with the reported AA simulation results in which limited improvement or even deterioration were observed on the transverse elastic modulus of CNT/epoxy nanocomposites [44].

In Section 3.2.1, although the radial opening mode simulations show that the interface load transfer predicted by the CG models was underestimated, this deviation shows a negligible effect on the lateral elastic moduli. Since the aim of developing CG models is to reduce the complexity of their AA counterparts while retaining all the most important features, we also chose λ=5 Å. There is a price we have to pay for developing an efficient but simple CG model.

**Figure 8:**
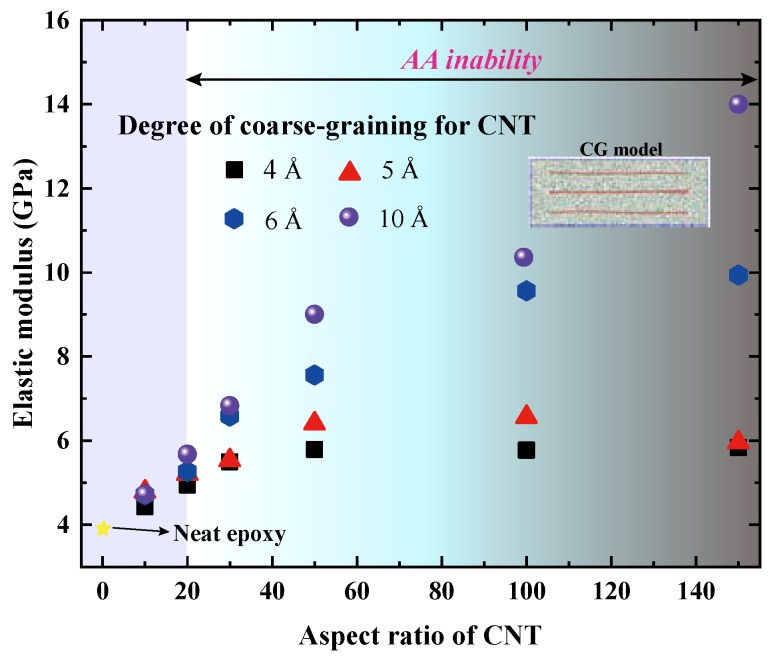
Axial elastic moduli of epoxy-matrix nanocomposites reinforced by the CNTs which have different aspect ratios and various degrees of coarse-graining.

We next evaluate the tensile responses along the longitudinal (axial) direction of the embedded CNTs, which are illustrated in Figure 8. Different from what we observed in Figure 7, the adopted λ has a significant effect on the axial elastic modulus ($E_{axial}$) of the CNT/epoxy nanocomposites. As shown in Figure 8, for each fixed λ value, $E_{axial}$ increases with the increasing CNT aspect ratio in a nonlinear fashion and tends to be plateau values in the high aspect ratio regimes. In particular, the critical aspect ratio where $E_{axial}$ starts entering the plateau stage appears to be larger for higher λ. For instance, the critical aspect ratio increases from 50 to 100 when λ increases from 5 to 6 Å. When λ is increased to 10 Å, the measured $E_{axial}$ is even not converged at the aspect ratio of 150 (CG models with a larger aspect ratio of the CNT were not carried out for the sake of computational resource). For each studied aspect ratio, the measured $E_{axial}$ of the CG models increases with the increasing value of λ and the deviation become larger at the cases of higher CNT aspect ratios. For instance, the calculated $E_{axial}$ for λ-10-10 (4.725 GPa) is only slightly larger than that of λ-4-10 (4.427 GPa). However, the $E_{axial}$ of λ-10-150 (14 GPa) is almost three times that of λ-4-150 (5.83 GPa). This phenomenon is attributed to the higher interface load transfer along the longitudinal direction when a larger λ was adopted. The better interface load transfer ability generally enables higher stress transfer from the matrix to CNT and thus leads to a larger tensile modulus of the CNT/epoxy nanocomposites [53,54]. As expected, $E_{axial}$ of CG models will be further increased if considering a larger λ.

The astonishing observations from Figure 8 strongly emphasize the importance of adopted λ in determining the studied tensile behavior of CNT/epoxy nanocomposites. According to the “monomeric separation response” analysis shown in Section 3.2, λ = 5 Å might be the most successful of the many values to the reproduction of the AA interface load transfer. As a result, the nanocomposites CG models with λ = 5 Å appears to be a suitable choice among the studied cases to capture the actual mechanical properties of CNT/epoxy nanocomposites.

Prior to this work, the most successful approaches in characterizing the CNT reinforcing efficiency in terms of different CNTs lengths use finite element analysis (FEA) or multi-scale modeling (such as the shear lag model, Halpin-Tsai, Mori-Tanaka) [53,54,55,56,57]. The tensile modulus predicted by these approaches typically converges almost to the rule of mixtures, which is much more larger than that calculated by our CG models [53]. This phenomenon is attributed to the unrealistic interactions of the CNT/matrix interface for these multi-scale modeling techniques which do not capture the weakly nonbonded vdWl nature of the interface. Moreover, CNTs with a high aspect ratio are assumed to be straight in both FEA and multi-scale modeling, which is not the real situation in the synthesized CNT/epoxy nanocomposites [58]. In our CG simulations, despite the momentum control of each embedded CNT (done by using the fix momentum command in LAMMPS), the curvature of embedded CNTs can also be observed clearly (details are shown in Supplementary Section S4). As widely demonstrated by previous studies, the waviness of CNTs has an evident deterioration on the tensile modulus of nanocomposites [37,56] and thus leads to a smaller critical CNT aspect ratio where the convergence of tensile modulus occurs.

### 3.4. Determination of the Interface Force Fields

In previous sections, we have demonstrated the critical role of λ in affecting the CNT/epoxy interface load transfer capability and the determined mechanical property of CNT/epoxy nanocomposites. In the principle of preserving the actual (AA model) interface behavior, λ = 5 Å appears to be reasonable in developing the CG models for CNT/epoxy nanocomposites. For CG models using λ = 5 Å, the cohesive interaction strength parameter $\varepsilon_{interface}$ is determined to be 1.849 kcal/mol. This value is, however, determined for the CNT/epoxy nanocomposites whose DOC of epoxy matrix is 90%. Besides, the simulation temperature is 300 K. Thus, whether the LJ parameters derived from DOC-90 and temperature 300 K would work at other DOC and temperatures remains to be validated.

**Figure 9:**
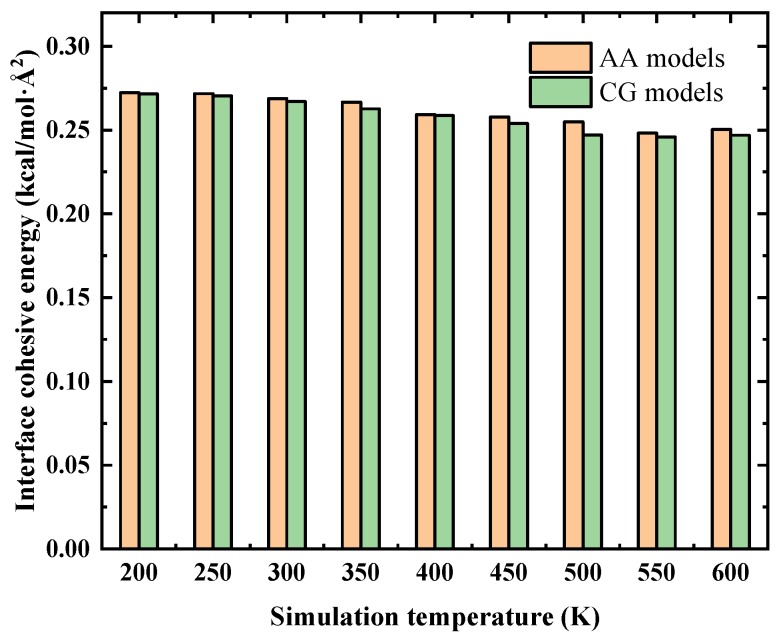
The developed CG models for CNT/epoxy nanocomposites work well over a wide temperature range.

To answer this question, we proceeded to evaluate the temperature issue of the developed CG models. As concluded from the previous sections, for a fixed λ, the interface load transfer ability is solely dependent on the interface cohesive energy. In other word, the evaluation of whether the CG force fields are able to work at other temperatures can be done through comparing the interface cohesive energy between the AA and CG models at various simulation temperatures. Figure 9 shows the comparison of interface cohesive energy between AA and CG models where the DOC is 90%. As temperature increases, the interface cohesive energy decreases for both AA and CG models. This phenomenon is originated from the reduced number of matrix molecules surrounding the CNT surface at higher temperatures because the matrix density generally decreases as temperature increases. Moreover, the deviation of interface cohesive energy between the AA and CG models at each studied temperature is slight (relative error less than 2%), indicating that the developed CG force fields of CNT/epoxy nanocomposites generally work well over a wide temperature range. It should be stressed here that the CG models of CNT/epoxy nanocomposites which performed well at different temperatures is a “set” of CG potentials because the potentials for cross-linked epoxies change at varies temperatures (see Supplementary Section S2.1 for details).

We next examine the DOC issue for the CG force fields through considering another 3 different matrix-DOC, that is, DOC-60, DOC-70, and DOC-80. The simulation temperature was kept at 300 K. Table 1 lists the values of the interface cohesive energy determined by the AA and CG simulations for the studied nanocomposites with 4 different values of DOC. The first thing we can observe is that, for AA models, the interface cohesive energy at various DOC are almost identical, which is mainly attributed to the insignificant effects of DOC on the density of the CNT/epoxy nanocomposites (1.198 ${g/\mathrm{cm}}^{3}$ for DOC-60 nanocomposites, and 1.206 ${g/\mathrm{cm}}^{3}$ for DOC-90 nanocomposites). However, for CG models, the interface cohesive energy was underestimated for DOC-60, DOC-70, and DOC-80 by the CG force fields derived from DOC-90. This observation strongly emphasizes that the CG force fields derived from DOC-90 are unable to transfer to other DOC. Different from the AA models, the DOC of matrix exhibits much more significant influence on the density (1.078 and 1.211 ${g/\mathrm{cm}}^{3}$ for DOC-60 and DOC-90 cross-linked epoxy, respectively) [23]. However, this effect on the interface cohesive energy of the nanocomposites CG models could be compensated for by adjusting the cohesive interaction strength parameter of the interface LJ potentials (see Section 3.1 for details). By phenomenologically preserving the AA interface cohesive energy, we determined a DOC-dependent $\varepsilon_{interface}$ for the interface LJ potentials of the CG models, yielding values of 2.331, 2.167, and 2.019 kcal/mol for DOC-60, DOC-70, and DOC-80 CNT/epoxy nanocomposites, respectively. The adjusted $\varepsilon_{interface}$ leads to a linear variation with the change of DOC, following the expression:

(5) $$\varepsilon_{interface}=3.287-1.594\times \mathrm{DOC}$$

Using these adjusted $\varepsilon_{interface}$, the interface cohesive energy of CNT/epoxy nanocomposites at various values of DOC are well preserved, as listed in Table 1.

After showing that the CG models for CNT/epoxy nanocomposites can also work well at different temperatures and DOC through using DOC- and temperature-dependent cohesive interaction strength ϵ for both epoxy matrix and the CNT/epoxy interface interactions, we provide here some particular notes for the practical use of the developed CG potentials.

- For the reinforcement force fields, the selection of degree of coarse-graining for (5,5) CNT should be ∼5 Å, which provides a faithful approximation to the interface load transfer characteristics of the AA models (see potential parameters for CNT (λ = 5 Å) in Supplementary Table S2).
- The cross-linked epoxy matrix force fields are based on our previous work and are shown in Supplementary Table S1 [23].
- The interface force field is modeled using the 12-6 LJ potentials where the cohesive interaction strength parameter ε is determined by Equation (5) (case of λ = 5 Å), whereas the length scale parameter σ is calculated as Equation (4).

## 4. Conclusions

In summary, we demonstrated for the first time that the interface characteristics of CG models for the CNTs/epoxy nanocomposites are generally inconsistent with their AA counterparts, causing artifact to the predicted mechanical properties. On one hand, the interface cohesive energies of the CG models are significantly higher than that of their AA counterparts, which will inevitably lead to an overestimated interface load transfer ability. On the other hand, the degree of coarse-graining λ of CNT plays a crucial role in affecting the interface load transfer behavior of the CG models. In particular, the peak sliding force for a single DGEBA molecule onto the CNT surface with λ = 10 Å can reach 1.37 nN, which is nearly 2 orders of magnitude higher than that of the real situation derived from the AA model (0.0354 nN). As a result, the predicted mechanical property of nanocomposites shows a very large deviation when various values of λ were adopted for developing the CG models.

To correctly reproduce the real interface (the AA system), our data strongly advocate two design criteria for coarse-grain modeling the CNTs/epoxy nanocomposites—(1) phenomenologically adjusting the cohesive interaction strength parameter ($\varepsilon_{interface}$) of the interface LJ potentials to preserve the interface cohesive energies of AA models and (2) carefully choosing a degree of coarse-graining for the embedded CNTs to capture the actual interface load transfer behavior derived from the AA models. We emphasize that only in this manner are the CG models of nanocomposites capable of providing useful insights into the predicted mechanical properties or mechanisms. 

## Figures and Tables

**Table 1:** Interface cohesive energy of CNT/epoxy nanocomposites using the AA and CG models.

	DOC-60	DOC-70	DOC-80	DOC-90
AA models	0.272	0.271	0.270	0.269
CG models	0.219	0.229	0.242	0.269
CG-adjusted	0.272	0.271	0.270	0.269

## Data Availability

The data that support our studies is available from the corresponding authors on reasonable request.

## Supplementary Materials

The following are available online at https://www.mdpi.com/2079-4991/9/10/1479/s1.
