# Supplementary material for: Importance of Interface in the Coarse-Grained Model of CNT /Epoxy Nanocomposites
Source: Nanomaterials (Basel). 2019 Oct 17;9(10):1479. doi: 10.3390/nano9101479 (PMC6835526; doi:10.3390/nano9101479)
Supplement: Supplementary file 1 [file nanomaterials-09-01479-s001.zip › SI.pdf]

nanomaterials

MDPI

Article

Importance of Interface in the Coarse-Grained Model of CNT /Epoxy Nanocomposites

Ke Duan [1], Li Li [2], Fei Wang [1], Weishuang Meng [1], Yujin Hu [1,*] and Xuelin Wang [1]

1 State Key Lab of Digital Manufacturing Equipment and Technology, School of Mechanical Science and Engineering, Huazhong University of Science and Technology, Wuhan 430074, China; duank@hust.edu.cn (K.D.); d201880192@hust.edu.cn (F.W.); m201770371@hust.edu.cn (W.M.); wangxl@hust.edu.cn (X.W.)

2 School of Mechanical and Aerospace Engineering, Nanyang Technological University, Nanyang Avenue, Singapore 639798, Singapore; lili_em@hust.edu.cn

* Correspondence: yjhu@mail.hust.edu.cn

Received: date; Accepted: date; Published: date

check for updates

Supporting Information

This PDF file includes:

Section S1. Equilibration procedures for all-atomistic and coarse-grained models

Section S2. CG force fields of (5,5) CNT and cross-linked epoxy

Section S3. Radial distribution function curves of all-atomistic and coarse-grained models

S1. Equilibration procedures for all-atomistic and coarse-grained models

S1.1. All-atomic model

The all-atomic (AA) model of CNT/epoxy nanocomposite consists of a (5,5) CNT with the length equal to 75.7 Å(periodically along the longitudinal direction) and 861 matrix molecules (including 574 DGEBA and 287 DDM molecules). The initial density of the AA model is around 0.5 g/cm^3 and has a total number of atoms equal to 37444. To relax the all-atomistic (AA) model, an energy minimization was initially carried out using the conjugate gradient algorithm with an energy tolerance of 10^{-8} kcal/mol. Subsequently, the simulation cell was equilibrated at a high temperature 1000 K under an isochoric and isothermal ensemble (NVT) for 500 ps in order to achieve a uniform and faster mixture process. After that, the density of simulation model was corrected through turning the ensemble to isothermal-isobaric (NPT) with a 1 atm pressure for 100 ps. Finally, the simulation model was slowly cooled down to a temperature 700 K within 500 ps and fully relaxed at this temperature for 1 ns. As mentioned in the main paper, the cross-linking reaction was performed at 700 K. After the epoxies were cross-linked to a target degree of cross-linking (DOC), the simulation cell necessitates another equilibrations to eliminate the high internal stress induced by cross-linking. Such equilibration involves an anneal stage which firstly increases temperature from 700 to 1000 K and then cooled down to 700 K within 1 ns. At last, the AA system was cooled down to a target temperature and maintained at this temperature for 1 ns. Figure S1(a) depicts the equilibrated AA model of CNT/epoxy nanocomposites at temperature 300 K. Nose-Hoover thermostat and barostat were adopted to control the temperature and pressure of the all-atomic simulation model. Periodic boundary conditions were imposed on all three directions of the simulation box, i.e. the x, y, and z directions. The used timestep for all AA simulations is 1 fs.

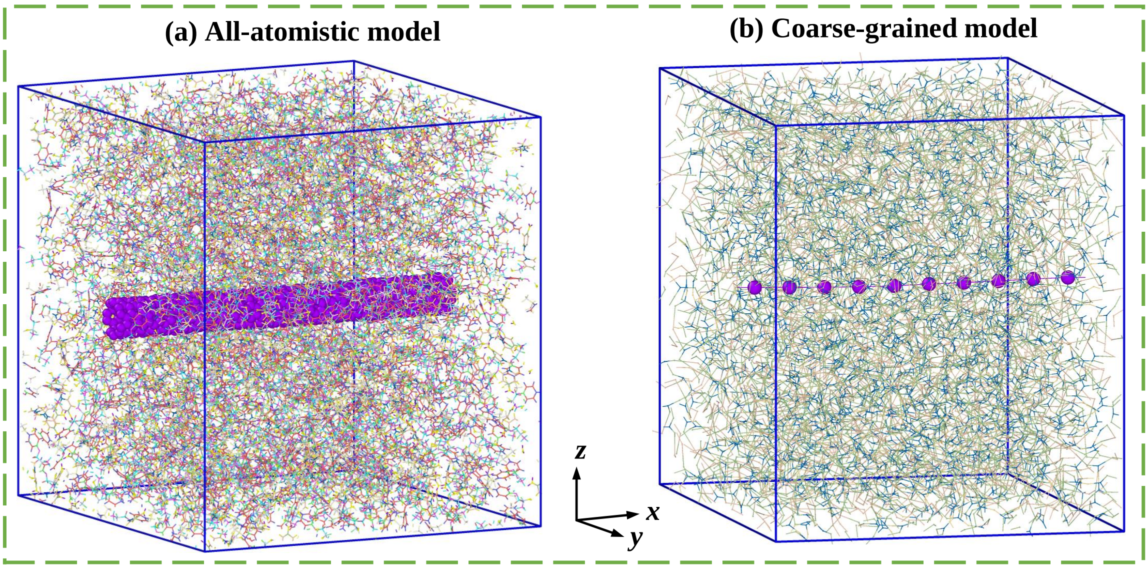

Figure S1. Simulation models for determining the interface cohesive energy for CNT/epoxy nanocomposites. (a) All-atomic model, and (b) Coarse-grained model (the degree of cross-linking λ for CNT is 10 Å).

S1.2. Coarse-grained model

Generally, in comparison with AA simulations, their coarse-grained (CG) counterparts are more computational efficient and therefore enable the construction of larger simulation models. The CG model of the CNT/epoxy nanocomposite consists of a 100-Å-long (5,5) CNT (periodically along the longitudinal direction) and 3294 matrix molecules (including 2196 DGEBA and 1098 DDM molecules)

with an initial density around 0.85 g/cm^3. The total number of CG particles is 9892. The inter-molecule interactions were described by the 12-6 LJ potential with a cutoff distance 30 Å. In comparison with AA simulations, the equilibration time of CG models can be more faster due to their reduced number of degrees of freedom and faster dynamic characteristics. Before the cross-linking process, the CG model was relaxed at 700 K under NVT ensemble for 1 ns followed by an equilibration procedure under NPT ensemble for another 1 ns with a 1 atm pressure. It should be mentioned that this relaxation process is sufficient to equilibrate the CG model and to achieve a uniform mixture of the matrix molecules. After the CG model was fully equilibrated, the cross-linking reaction was carried out at temperature 700 K using the dynamic cross-linking approach. After that, the CG model in which the epoxies were cross-linked to a target DOC was again relaxed to release the internal stress due to the cross-linking. This is done by performing an anneal procedure under NPT ensemble which firstly increases simulation temperature from 700 to 1000 K within 4 ns and then maintained at 1000 K for 24 ns. Subsequently, the CG model was cooled down to a target temperature within 8 ns and maintained at this temperature for another 16 ns. The temperature and pressure were controlled by Nose-Hoover thermostat and barostat with a timestep 4 fs. Similar to AA models, periodic boundary conditions were also applied to the three directions of CG simulation box. The equilibrated CG model for CNT/epoxy nanocomposite at temperature 300 K is shown in Figure S1(b).

S2. Coarse-grained force fields of (5,5) CNT and cross-linked epoxy

S2.1. Cross-linked epoxy

In this section, we present the coarse-grained (CG) force fields for both cross-linked epoxy and (5,5) CNT, which are determined individually. Table S1 illustrates the CG force field for cross-linked epoxy over wide ranges of degree of cross-linking and temperatures [1]. In corresponding to the mapping scheme shown in Figure 1 of the main paper, the bonded interactions include 3 bond types and 5 angle types, whereas the nonbonded interactions were described by using 12-6 LJ form with a uniform cohesive interaction strength parameter ϵ. Specifically, the LJ parameter ϵ is a function of both temperature and the degree of cross-linking (DOC) of epoxy, as depicted in equation (1). In equation (1), α and β are two fitting parameters related to DOC and have a function of equation (2) and equation (3), respectively. For more details, the reader can be directed to our previous work [1].

Table S1. CG force field for cross-linked epoxy over wide ranges of degrees of cross-linking and temperatures [1].

Interaction	Potential form	Type	Parameters
Bond	$U_{bond}(l) = k_{bond}(l - l_0)^2$	A-B	k_{AB}=13.103 kcal/(mol Å^2), l_0=6.889 Å
		A-C	k_{AC}=4.385 kcal/(mol Å^2), l_0=3.527 Å
		C-D	k_{CD}=40.515 kcal/(mol Å^2), l_0=3.856 Å
Angle	$U_{angle}(\theta) = k_\theta(\theta - \theta_0)^2$	A-B-A	k_{ABA}=5.540 kcal/(mol Å^2), θ_0=127.757°
		A-C-A	k_{ACA}=8.288 kcal/(mol Å^2), θ_0=97.692°
		A-C-D	k_{ACD}=2.902 kcal/(mol Å^2), θ_0=163.335°
		B-A-C	k_{BAC}=0.787 kcal/(mol Å^2), θ_0=147.971°
		C-D-C	k_{CDC}=31.886 kcal/(mol Å^2), θ_0=140.663°
Nonbond	$U_{nonbond} = 4\varepsilon\left[\left(\frac{\sigma}{r}\right)^{12} - \left(\frac{\sigma}{r}\right)^{6}\right]$		see ϵ in equation (1), σ_{AA}=5.4092 Å, σ_{BB}=6.5172 Å, σ_{CC}=5.7851 Å, σ_{DD}=5.7251 Å.

$$\varepsilon = \alpha\left(\frac{T}{300} - 1\right)^2 + \beta\left(\frac{T}{300} - 1\right) + 2.1566 \tag{1}$$

$$\alpha = -5.11983 \times \text{DOC}^2 + 3.92678 \times \text{DOC} + 0.67407 \tag{2}$$

$$\beta = 10.44367 \times \text{DOC}^2 - 12.13477 \times \text{DOC} + 1.52514 \tag{3}$$

S2.2. (5,5) carbon nanotube

The CG force fields for (5,5) CNTs based on the principle of force matching, which was first developed by Buehler [2]. As shown in Figure 1 of the main paper, the particle type for CNTs is labeled as 'G'. Thus, the bonded interactions include 1 bond type 'G G' and 1 angle type 'G G G'. The nonbonded van der Waals interaction between different CNTs are described with 12-6 LJ potential. In this work, the degree of coarse-graining λ of (5,5) CNT, namely the equilibrium distance between two 'G' particles, are ranged from 4 to 10 Å. The corresponding CG force fields are listed in Table S2.

Table S2. The CG force fields for (5,5) CNT with different values of λ.

λ (Å)	Bonded potentials		Nonbonded potential
	$U_{bond}(l) = k_{bond}(l - l_0)^2$	$U_{angle}(\theta) = k_\theta(\theta - \theta_0)^2$	$U_{nonbond} = 4\varepsilon\left[\left(\frac{\sigma}{r}\right)^{12} - \left(\frac{\sigma}{r}\right)^{6}\right]$
4	k_{bond}=1250 kcal/molÅ^2, l_0=4 Å	k_θ=17875 kcal/molÅ^2, θ_0=180°	ϵ=2.416 kcal/mol, σ=9.35 Å
5	k_{bond}=1000.0 kcal/molÅ^2, l_0=5 Å	k_θ=14300.0 kcal/molÅ^2, θ_0=180°	ϵ=3.775 kcal/mol, σ=9.35 Å
6	k_{bond}=833.5 kcal/molÅ^2, l_0=6 Å	k_θ=11916.5 kcal/molÅ^2, θ_0=180°	ϵ=5.436 kcal/mol, σ=9.35 Å
7	k_{bond}=714.3 kcal/molÅ^2, l_0=7 Å	k_θ=10214.3 kcal/molÅ^2, θ_0=180°	ϵ=7.399 kcal/mol, σ=9.35 Å
8	k_{bond}=625 kcal/molÅ^2, l_0=8 Å	k_θ=8937.5 kcal/molÅ^2, θ_0=180°	ϵ=9.664 kcal/mol, σ=9.35 Å
9	k_{bond}=555.6 kcal/molÅ^2, l_0=9 Å	k_θ=7944.4 kcal/molÅ^2, θ_0=180°	ϵ=12.231 kcal/mol, σ=9.35 Å
10	k_{bond}=500.0 kcal/molÅ^2, l_0=10 Å	k_θ=7150.0 kcal/molÅ^2, θ_0=180°	ϵ=15.1 kcal/mol, σ=9.35 Å

S3. Radial distribution function curves of all-atomistic and coarse-grained models

As described in Section 3.1 of the main paper, the interface interactions of the nanocomposites CG models are described using 12-6 LJ potentials, and consist of 4 LJ pairs, i.e., GA, GB, GC, and GD. The length scale parameter σ of these LJ pairs, which is the most critical parameter in affecting the distribution state of epoxy molecules around the CNT surface, is calculated using the expression $\sigma_{ij} = (\sigma_{ii} + \sigma_{jj})/2$, in which i represents the CNT particle 'G' and j denotes different particle types of epoxy (particle type A, B, C, and D).

To evaluate whether such distribution state is in well agreement with the real situation (AA system), we compare the radial distribution function (RDF) curves for the fully equilibrated AA and CG models. As shown in Figure S2, the relaxed AA trajectory (Figure S2(a)) was mapped to coarse-grained configuration (Figure S2(b)) using our in-house code according to the mapping scheme illustrated in Figure 1 of the main paper. Then, the radial distribution function (local density divide by overall density) of epoxy molecules, which is locating at a surface of CNT at the distance r, was calculated. The maximum r considered was 30 Å, and was divided into 60 small regions. The distribution state of these 4 epoxy beads for the AA and CG models are shown in Figure S3 and Figure S4, respectively.

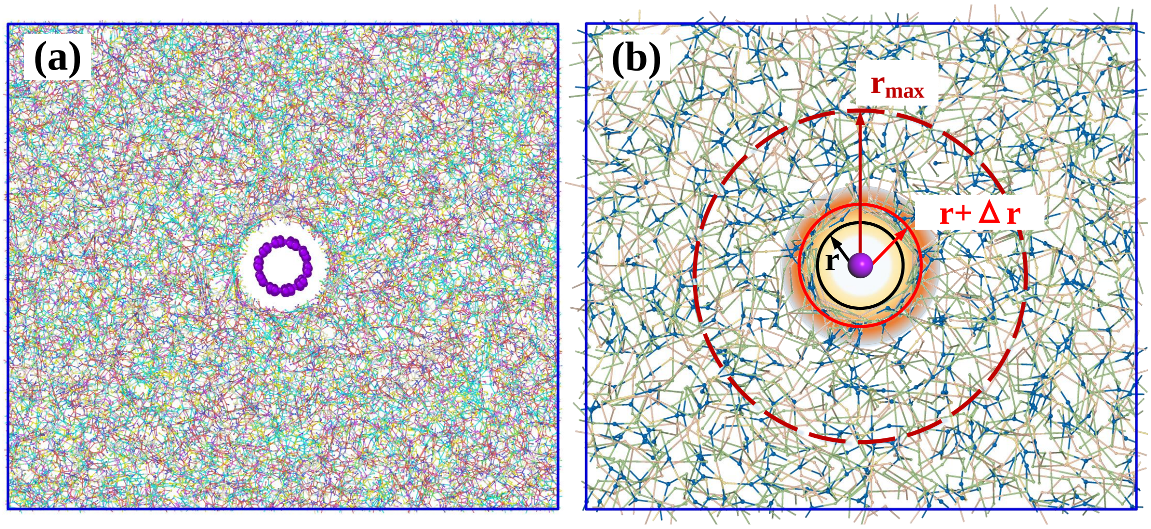

Figure S2. Atomic configuration of (a) the fully relaxed AA model of CNTs/epoxy nanocomposites, and (b) its CG counterpart and the illustration of how to determine the RDF curves.

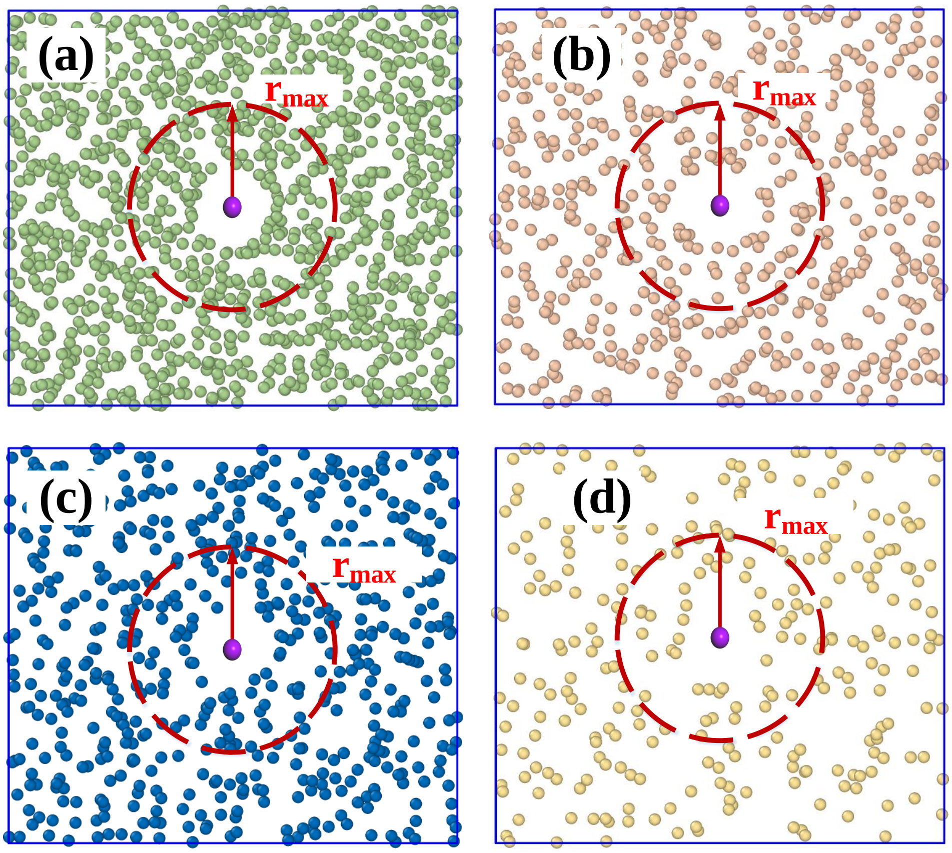

Figure S3. Distribution state of (a) 'A' particles, (b) 'B' particles, (c) 'C' particles, and (d) 'D' particles. It should be mentioned that these coarse-grained configurations were obtained by mapping the equilibrated AA model.

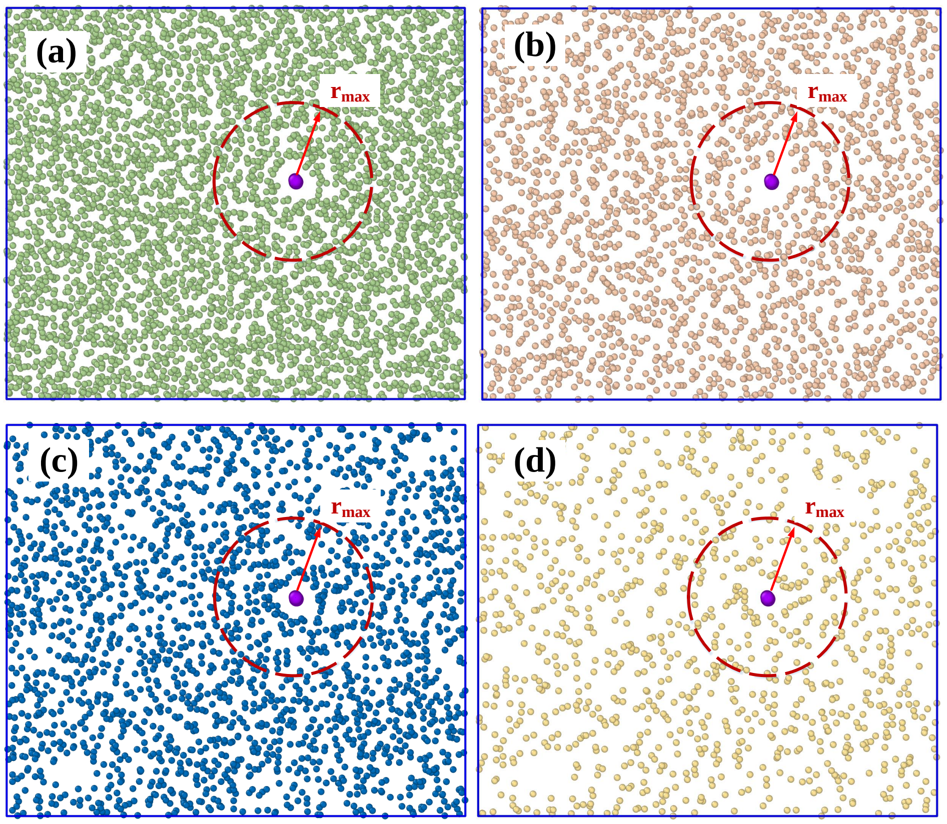

Figure S4. Distribution state of epoxy beads for CG model (a) 'A' particles, (b) 'B' particles, (c) 'C' particles, and (d) 'D' particles.

References

1. Duan, K.; He, Y.; Li, Y.; Liu, J.; Zhang, J.; Hu, Y.; Lin, R.; Wang, X.; Deng, W.; Li, L. Machine-learning assisted coarse-grained model for epoxies over wide ranges of temperatures and cross-linking degrees. *Mater. Des.* **2019**, *183*, 108130.
2. Buehler, J.M. Mesoscale modeling of mechanics of carbon nanotubes: Self-assembly, self-folding, and fracture. *J. Mater. Res.* **2006**, *21*, 2855–2869.

© 2019 by the authors. Licensee MDPI, Basel, Switzerland. This article is an open access article distributed under the terms and conditions of the Creative Commons Attribution (CC BY) license (http://creativecommons.org/licenses/by/4.0/).
