# Supplementary figures and images for: Importance of Interface in the Coarse-Grained Model of CNT /Epoxy Nanocomposites
Source: Nanomaterials (Basel). 2019 Oct 17;9(10):1479. doi: 10.3390/nano9101479 (PMC6835526; doi:10.3390/nano9101479)

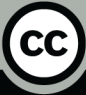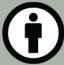

BY

Supplement: Supplementary file 1 [file nanomaterials-09-01479-s001.zip › Definitions/logo-ccby-eps-converted-to.pdf]

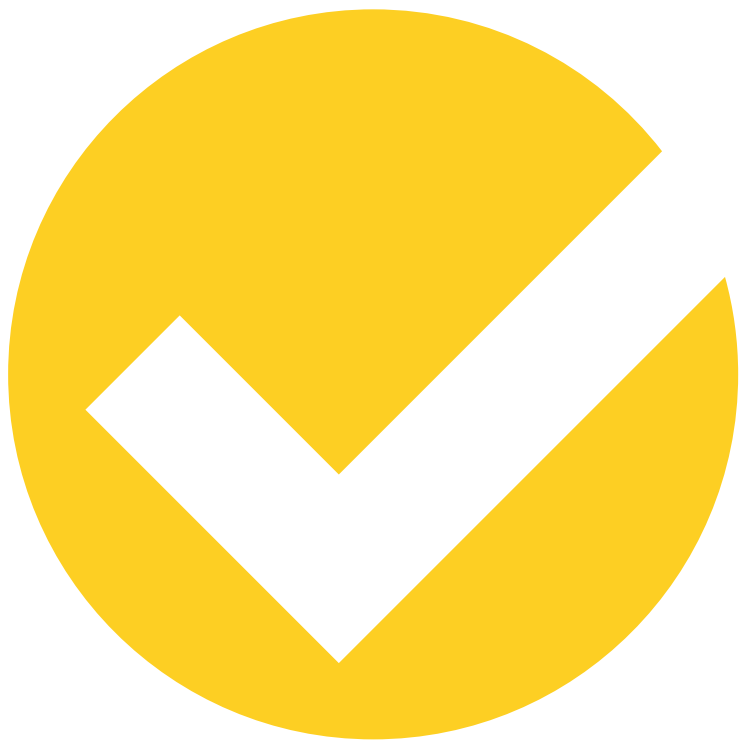

check for  
updates

Supplement: Supplementary file 1 [file nanomaterials-09-01479-s001.zip › Definitions/logo-updates.pdf]

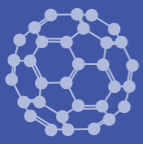

*nanomaterials*

Supplement: Supplementary file 1 [file nanomaterials-09-01479-s001.zip › Definitions/nanomaterials-logo-eps-converted-to.pdf]

**(a) All-atomistic model**

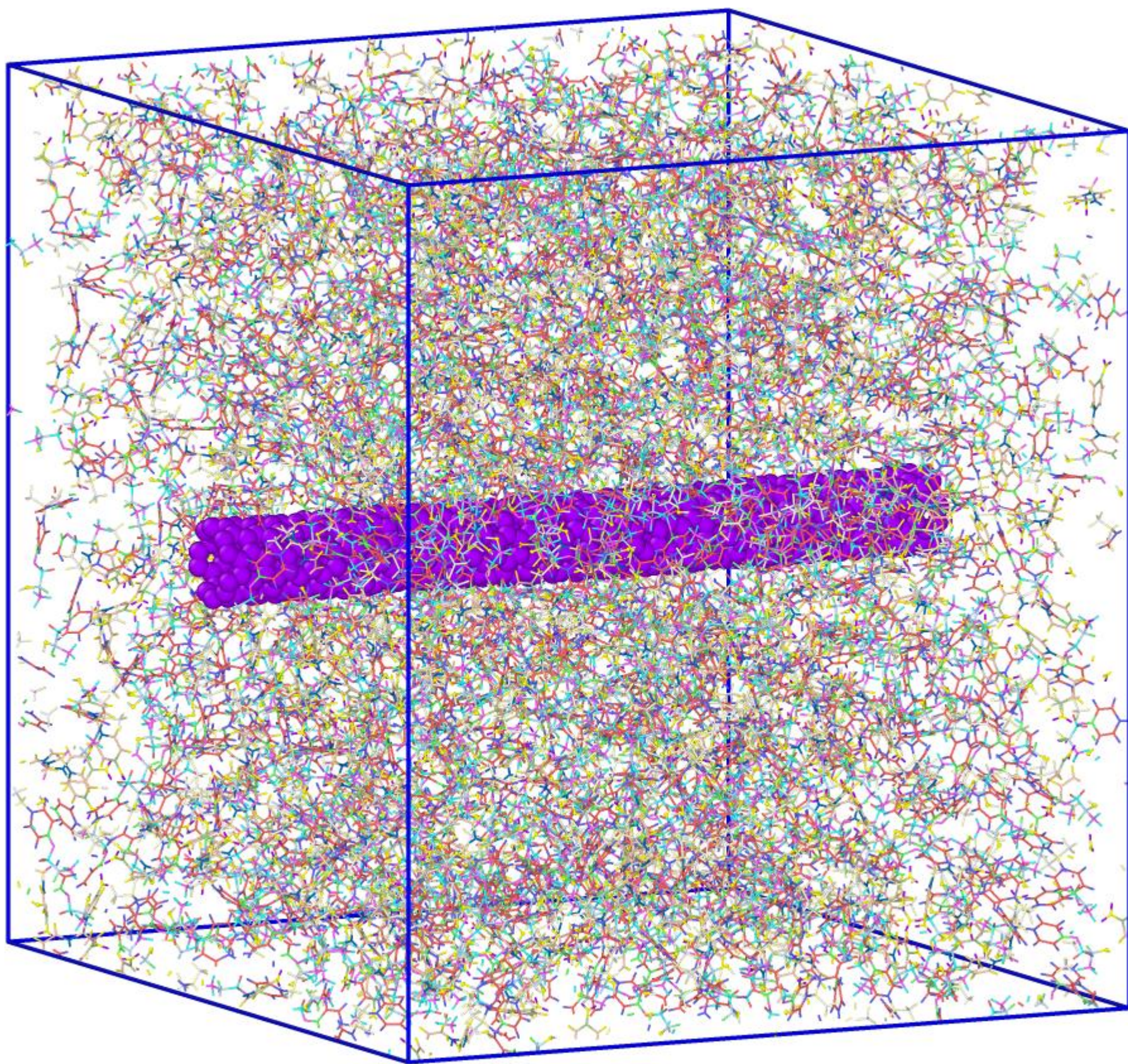

**(b) Coarse-grained model**

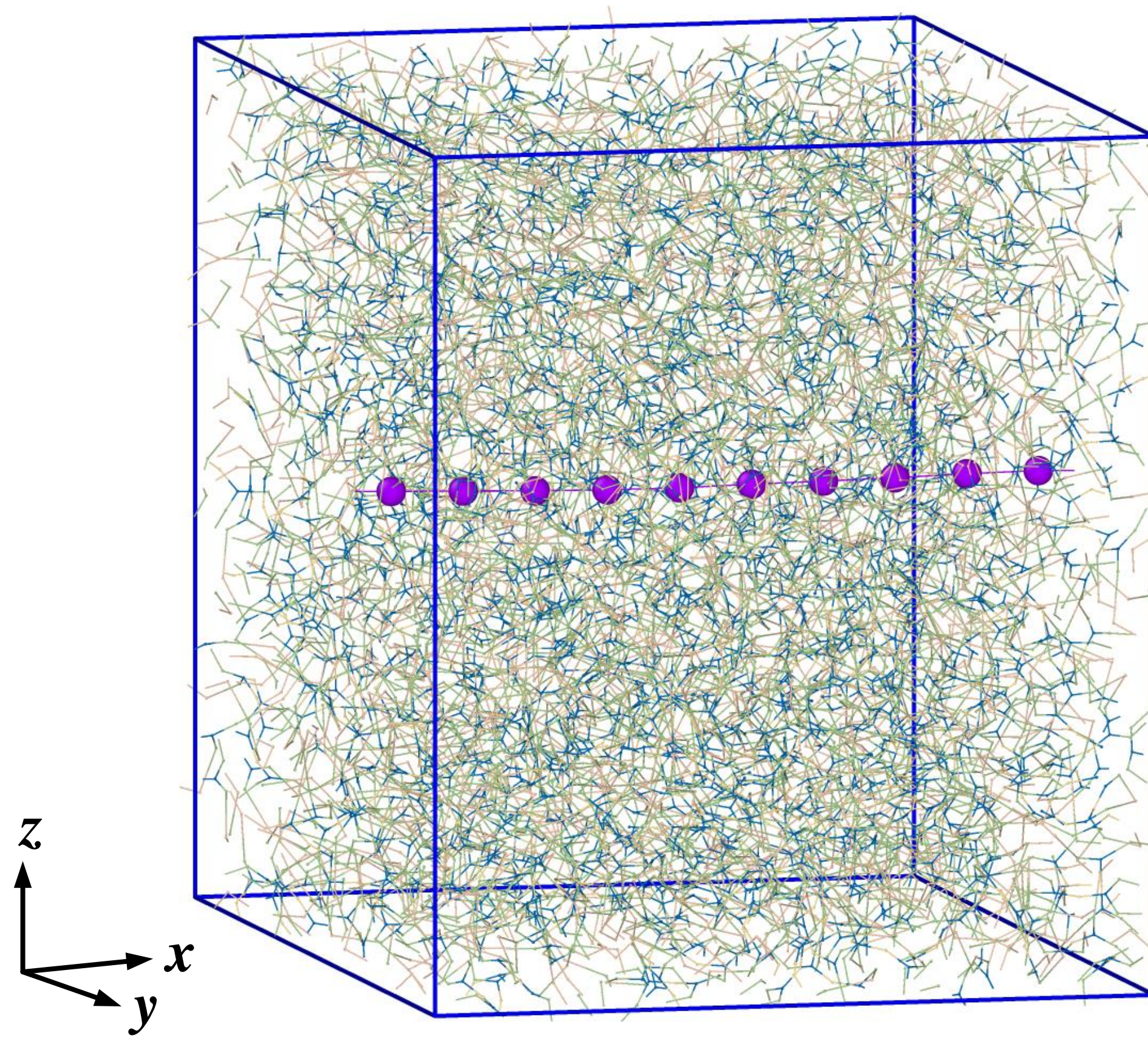

Supplement: Supplementary file 1 [file nanomaterials-09-01479-s001.zip › Figures/FigureS1.pdf]

**(a)**

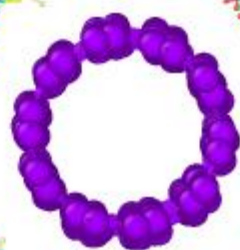

**(b)**

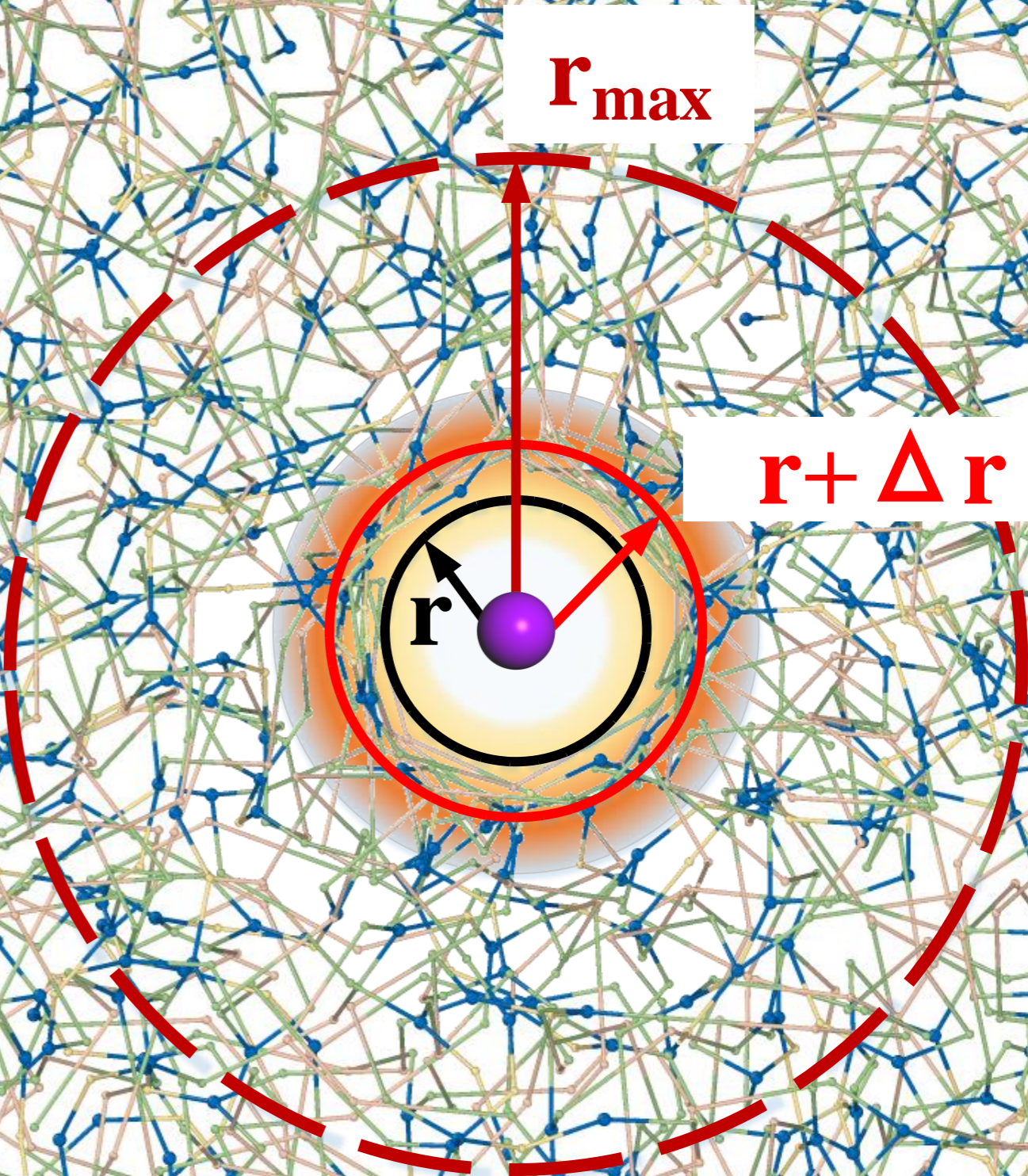

Supplement: Supplementary file 1 [file nanomaterials-09-01479-s001.zip › Figures/FigureS2.pdf]

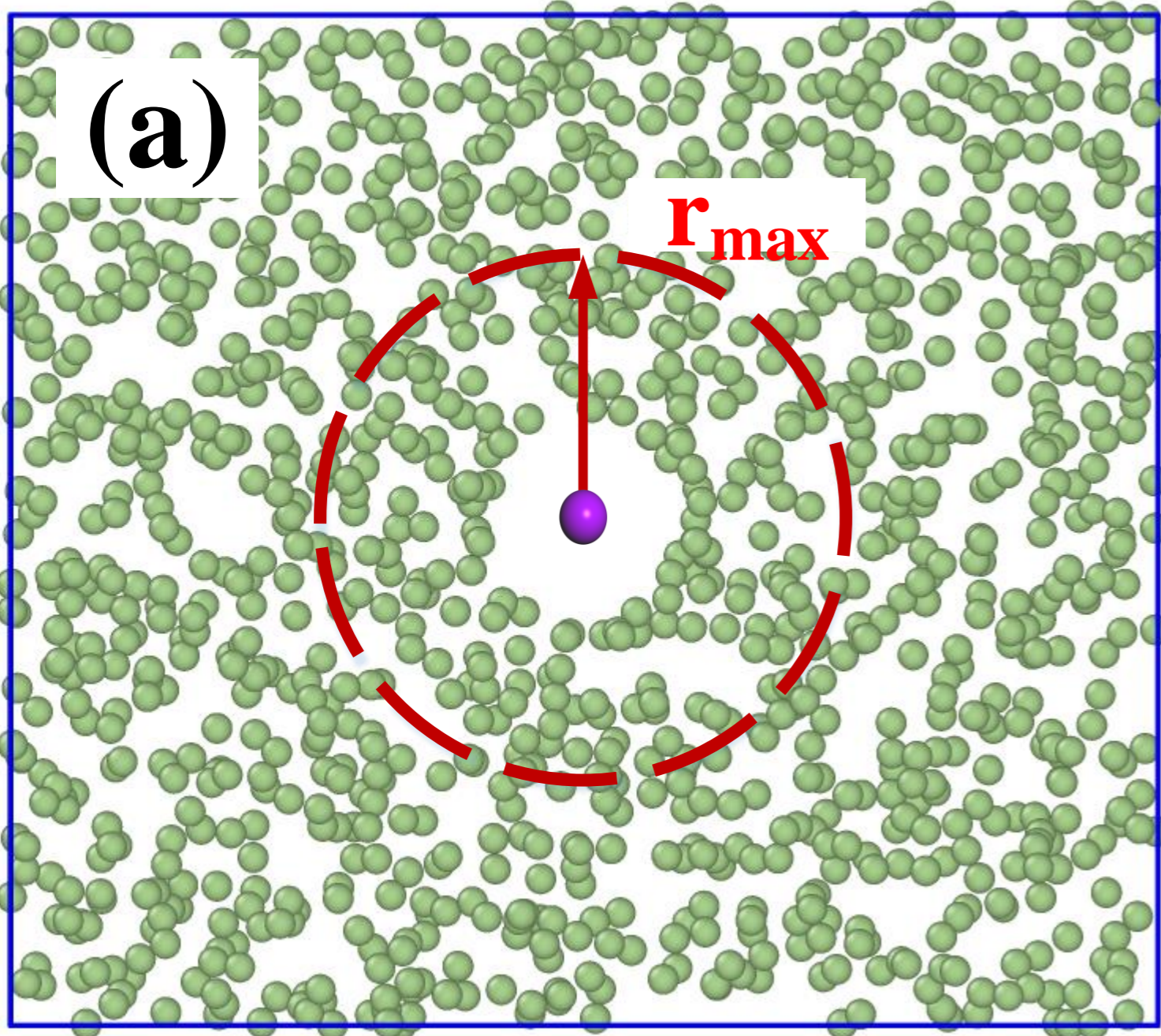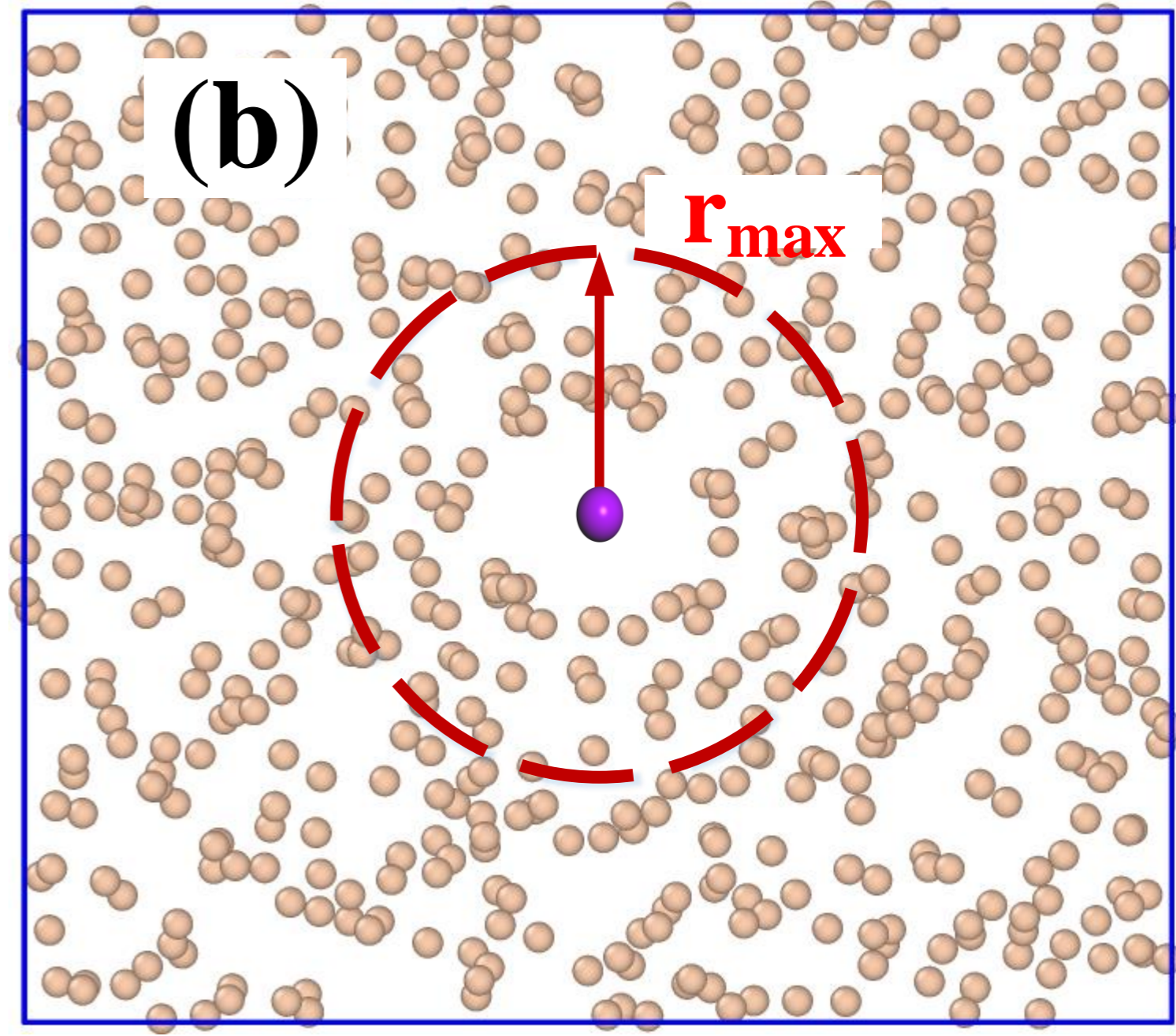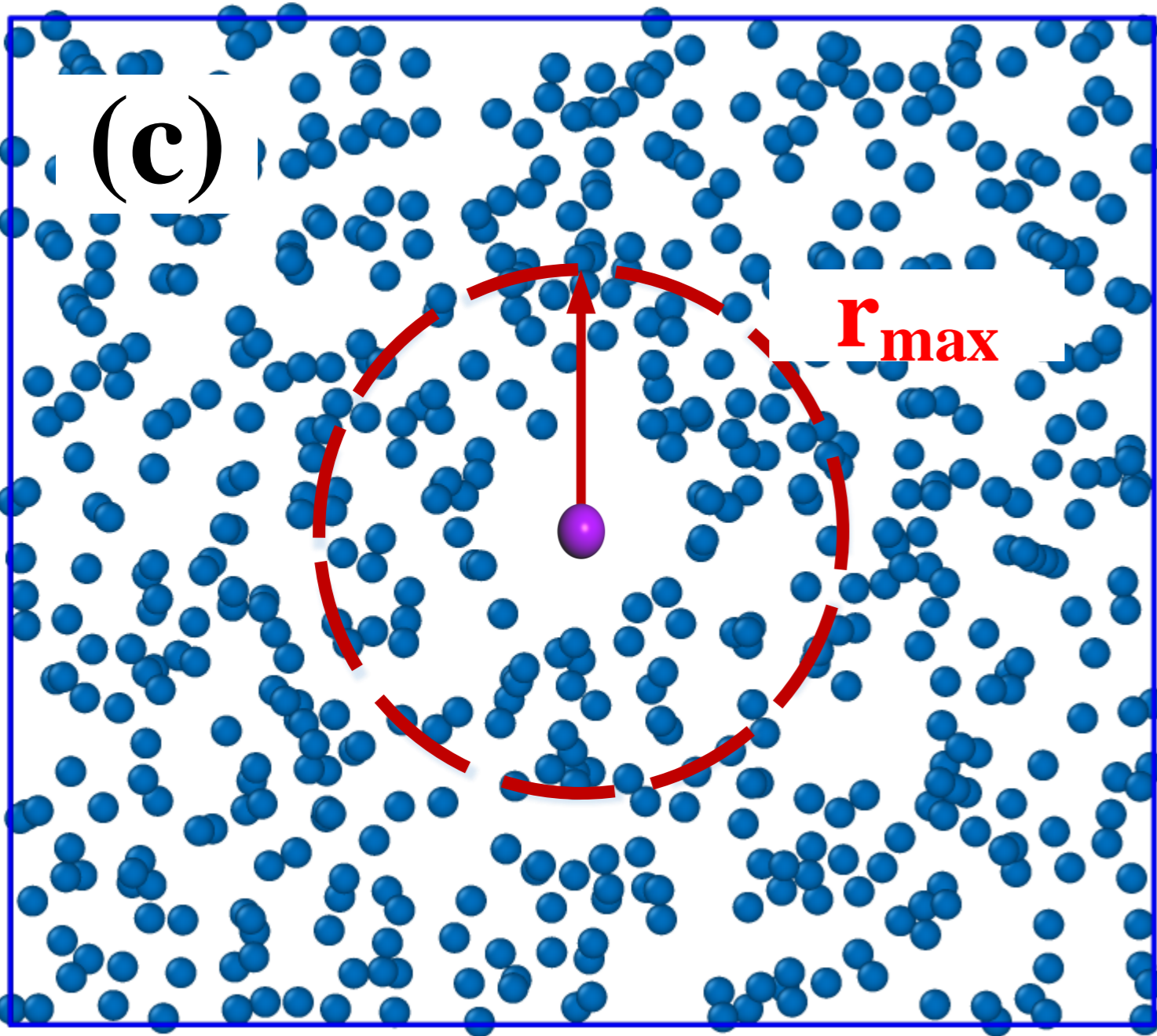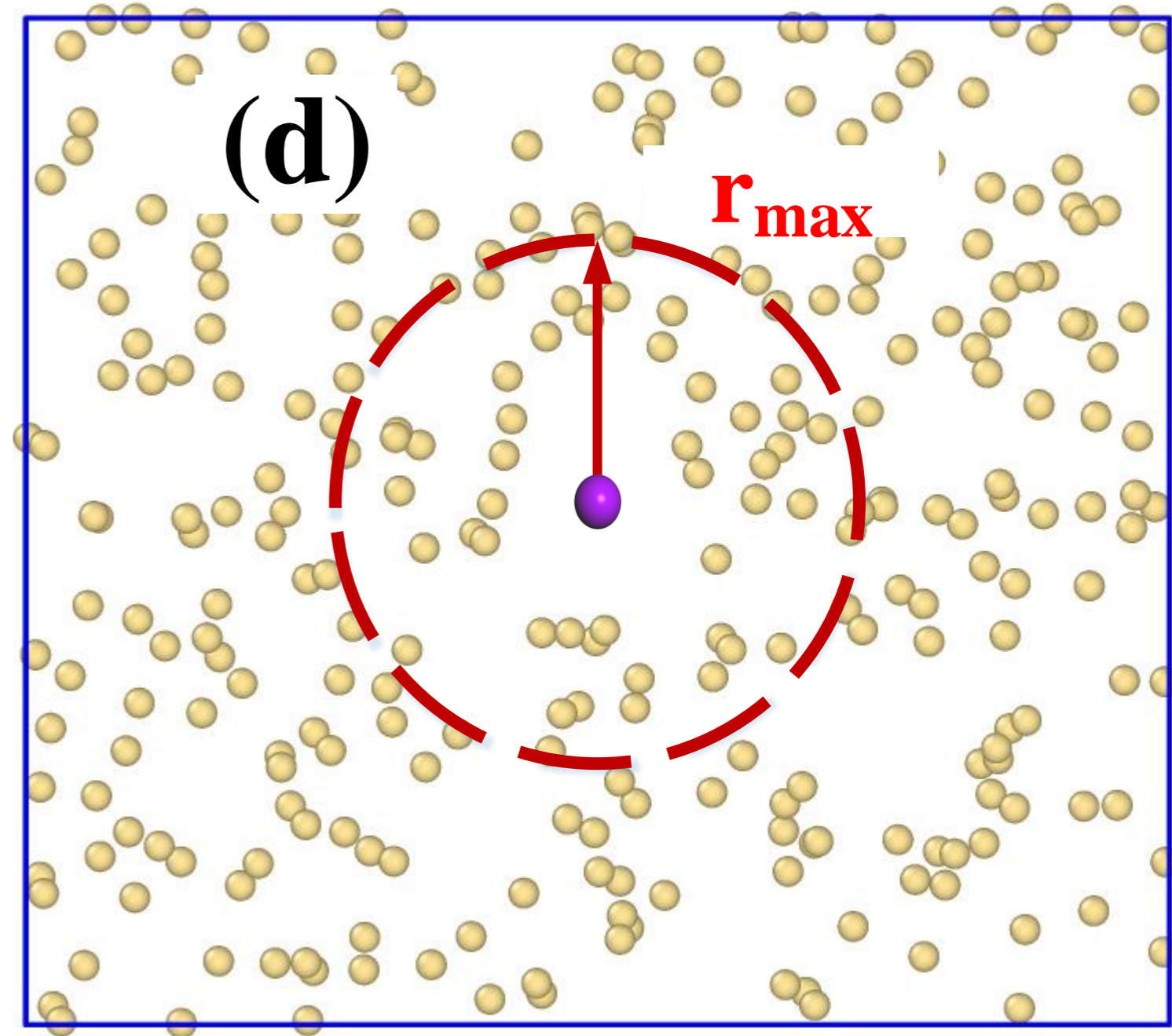

Supplement: Supplementary file 1 [file nanomaterials-09-01479-s001.zip › Figures/FigureS3.pdf]

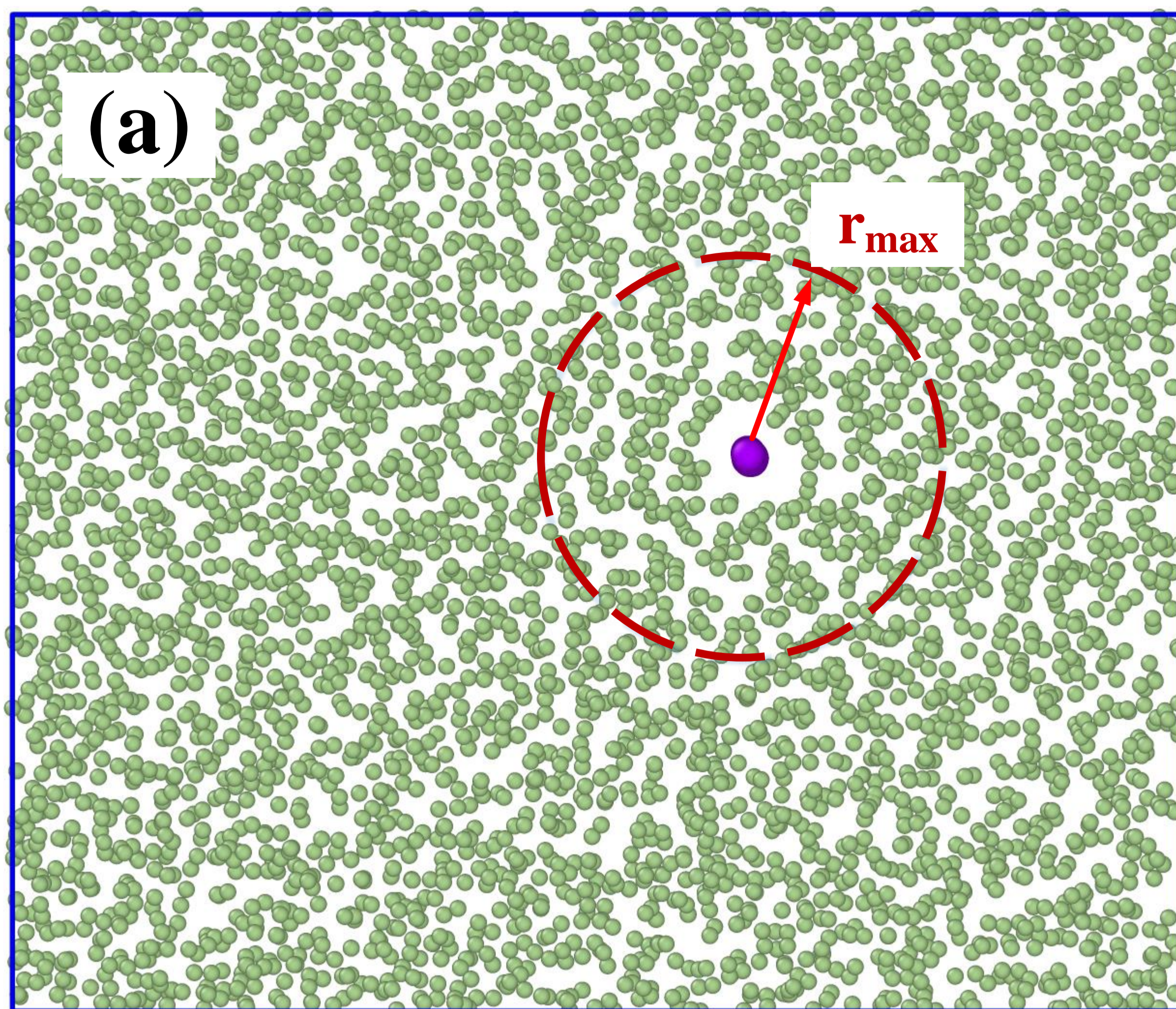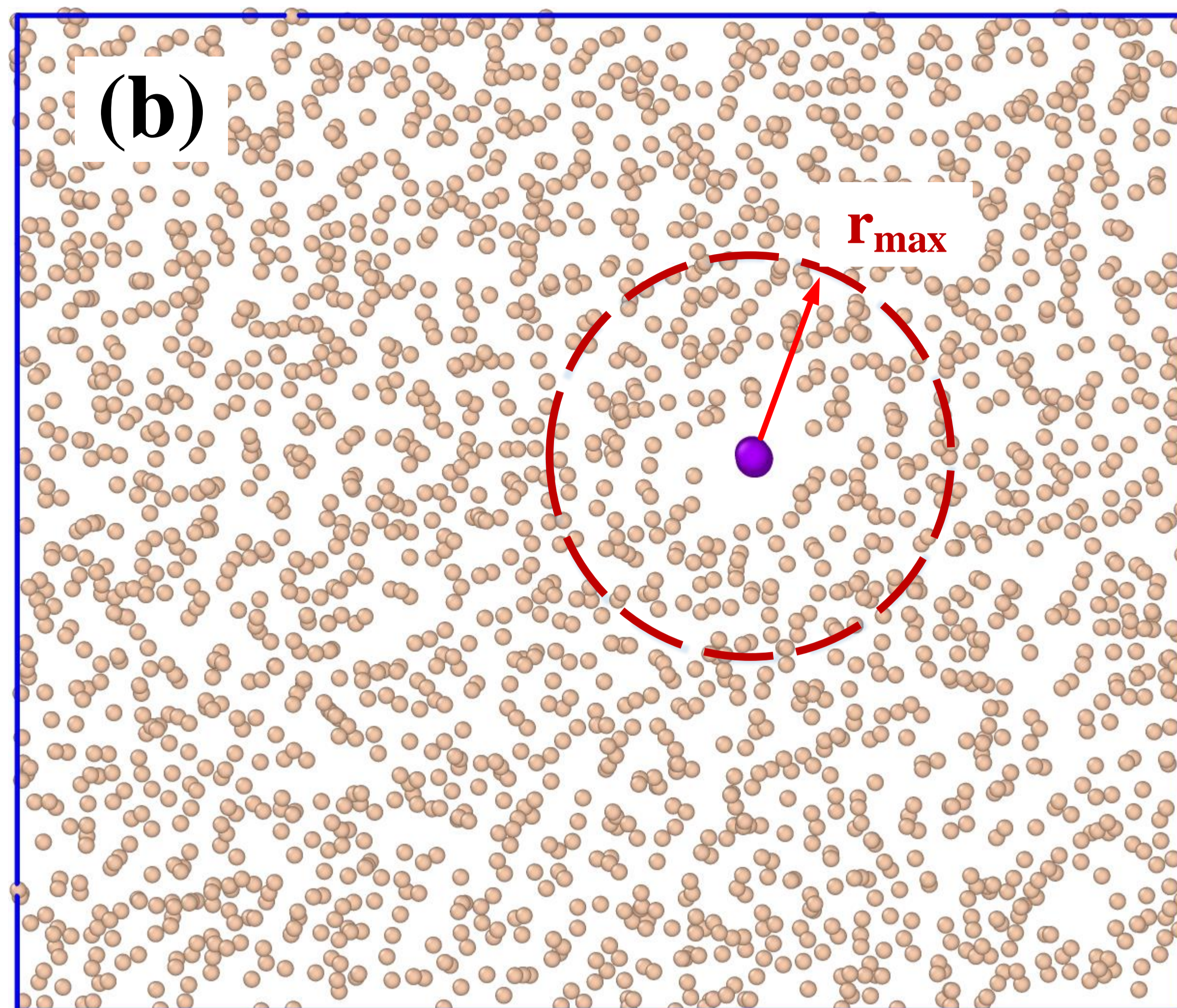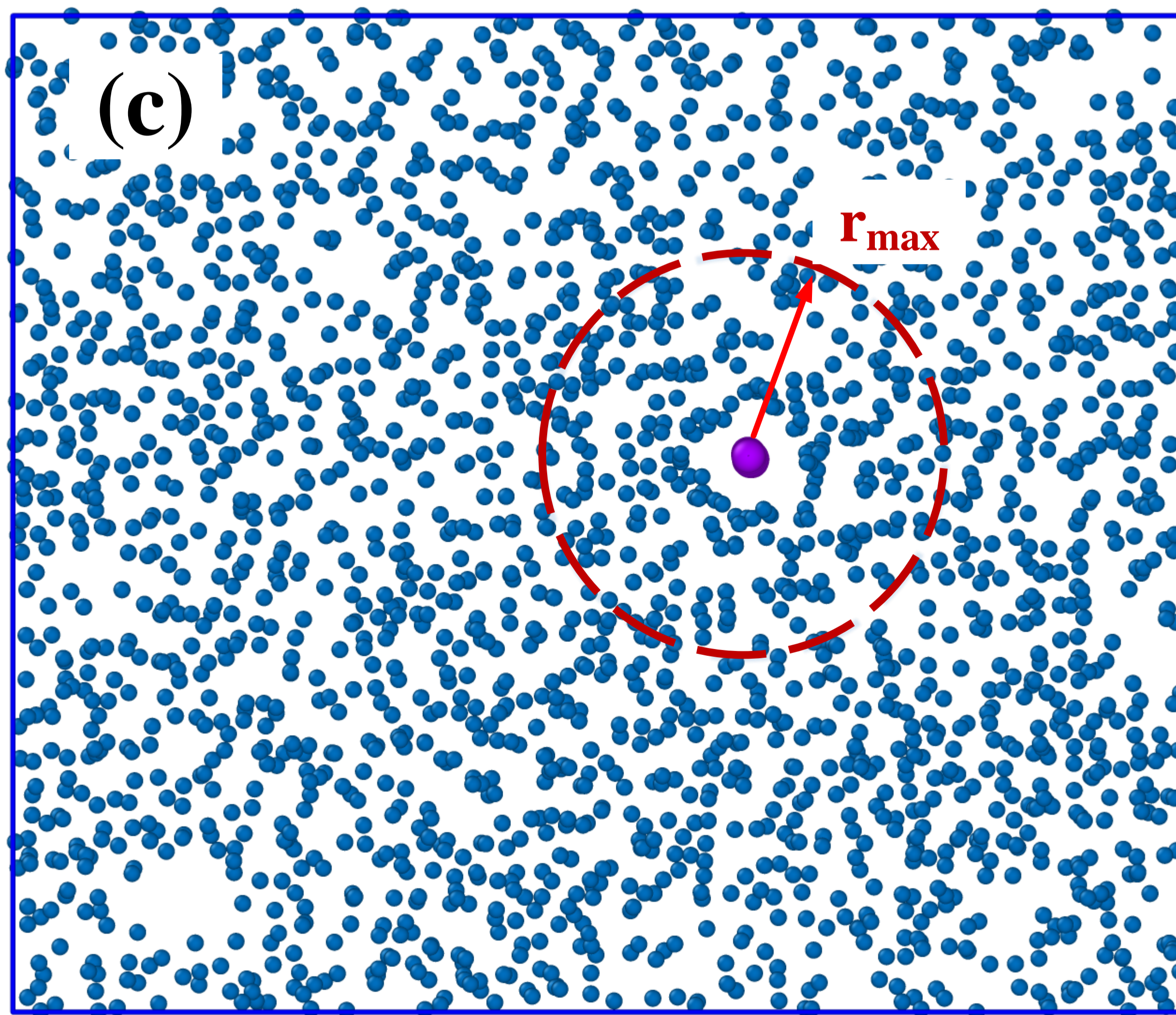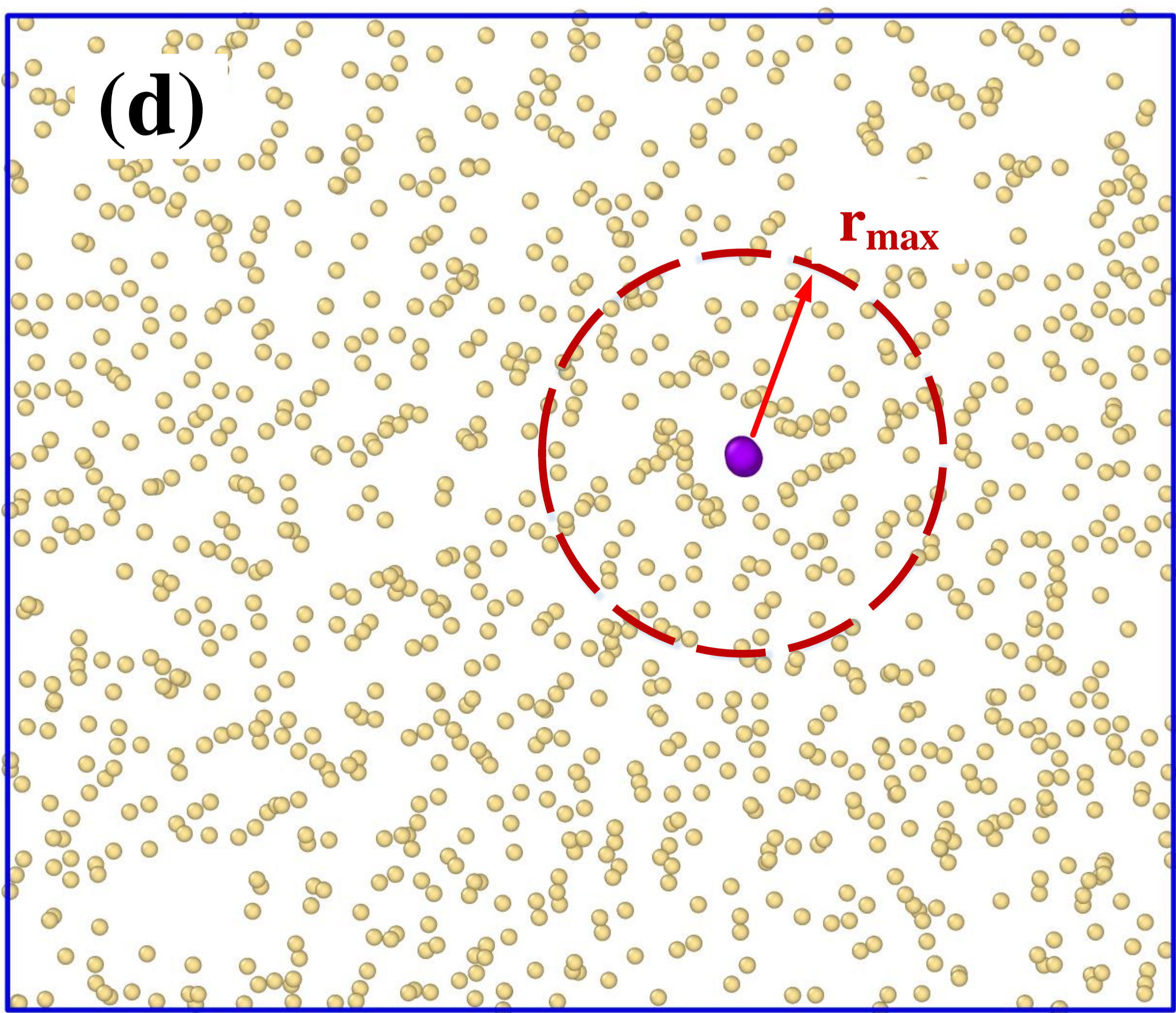

Supplement: Supplementary file 1 [file nanomaterials-09-01479-s001.zip › Figures/FigureS4.pdf]
